# Fabrication and Actuation of Magnetic Shape-Memory Materials

**DOI:** 10.1021/acsami.3c14091

**Published:** 2023-11-04

**Authors:** Francisco J. Vazquez-Perez, Cristina Gila-Vilchez, Alberto Leon-Cecilla, Luis Álvarez de Cienfuegos, Dmitry Borin, Stefan Odenbach, James E. Martin, Modesto T. Lopez-Lopez

**Affiliations:** † Departamento de Física Aplicada, Universidad de Granada, C.U. Fuentenueva, Granada E-18071, Spain; ‡ Instituto de Investigación Biosanitaria ibs.GRANADA, Avda. de Madrid 15, Granada E-18012, Spain; § Departamento de Química Orgánica, Unidad de Excelencia Química Aplicada a Biomedicina y Medioambiente, Universidad de Granada, C. U. Fuentenueva, Granada E-18071, Spain; ∥ Chair of Magnetofluiddynamics, Measuring and Automation Technology, Technische Universität Dresden, George-Bähr-Strasse 3, Dresden 01069, Germany; ⊥ Sandia National Laboratories, Albuquerque, New Mexico 87059, United States

**Keywords:** soft actuators, hydrogel machines, magnetic actuators, magnetic particles, biopolymers

## Abstract

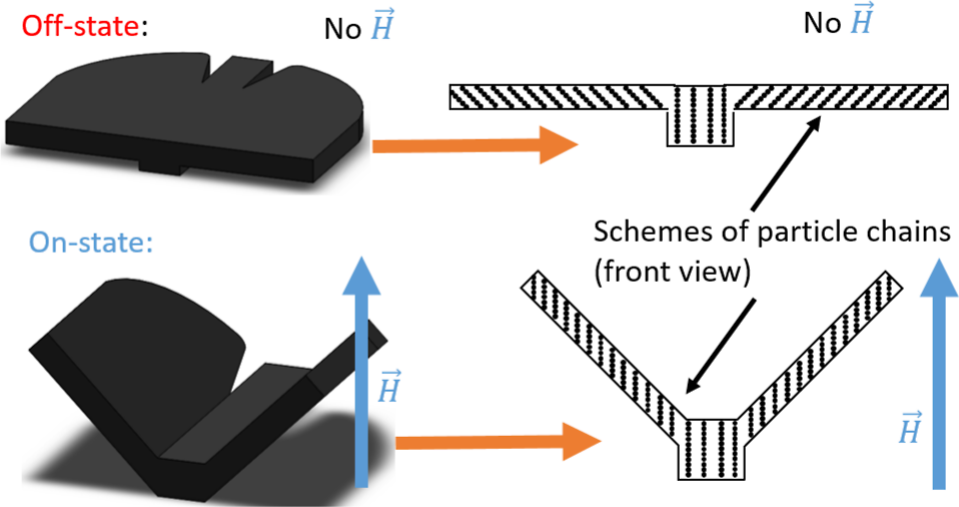

Soft actuators are deformable materials that change their dimensions or shape in response to external stimuli. Among the various stimuli, remote magnetic fields are one of the most attractive forms of actuation, due to their ease of use, fast response, and safety in biological systems. Composites of magnetic particles with polymer matrices are the most common materials for magnetic soft actuators. In this paper, we demonstrate the fabrication and actuation of magnetic shape-memory materials based on hydrogels containing field-structured magnetic particles. These actuators are formed by placing the pregel dispersion into a mold of the desired on-field shape and exposing it to a homogeneous magnetic field until the gel point is reached. At this point, the material may be removed from the mold and fully gelled in the desired off-field shape. The resultant magnetic shape-memory material then transitions between these two shapes when it is subjected to successive cycles of a homogeneous magnetic field, acting as a large deformation actuator. For actuators that are planar in the off-field state, this can result in significant bending to return to the on-field state. In addition, it is possible to make shape-memory materials that twist under the application of a magnetic field. For these torsional actuators, both experimental and theoretical results are given.

## Introduction

Soft robotics is a field of robotics that is based on the use of easily deformable, mechanically resilient materials intended for a variety of applications, such as soft grippers and artificial muscles.^1−8^ Unlike their rigid counterparts, soft robotic materials are intrinsically safe for contact use with humans such as in medical devices. These soft actuators can change dimensions and/or shape and can undergo locomotion, in response to stimuli such as pH, light, heat, solvent, electric or magnetic fields, etc.^9−19^ Among these stimuli, applied magnetic fields are one of the most attractive ways of actuation, due to their ease of application, prompt response, and safety in biological systems.^20−25^

Active materials are a rapidly emerging area of research interest, with approaches that include Marangoni propulsion,^26^ moisture-sensitive surface friction changes and actuation,^27−29^ electrostatic and thermal actuation,^30^ as well as actuation by combined responsiveness to light and magnetic field.^31^ Metachronal waves of artificial magnetic cilia have been created^32,33^ that can be controlled by a simple rotating magnetic field, and patterned magnetically anisotropic hydrogels have been developed^34^ based on the well-documented tendency of magnetic particles to form sheets in a rotating field.^35^

Soft magnetic actuators are usually made by dispersing magnetic particles in a polymeric network, either an elastomer or a gel, to form magnetic elastomers or magnetic gels. The use of elastomers is more common, although soft magnetic actuators based on hydrogels are of increasing interest due to their greater biocompatibility.^36−41^ Regardless of the type of polymer network, a strong, repeatable, and durable response to a magnetic field is essential. The current challenge is to develop a simple fabrication method that enables the preparation of soft magnetic actuators based on biocompatible compounds that can exhibit complex three-dimensional (3D) movements.

**Figure 1:**
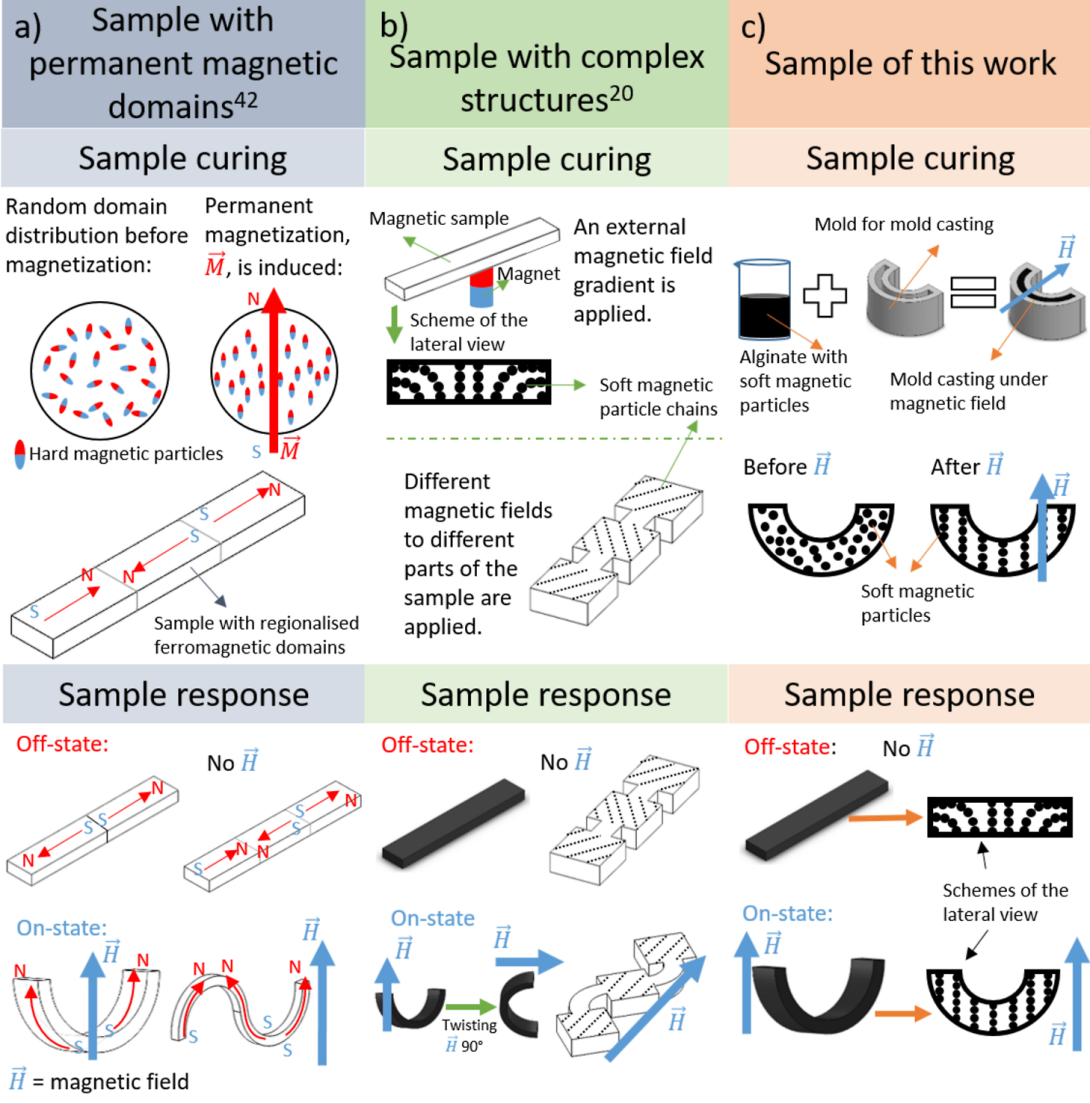
Scheme of different ways to prepare soft magnetic responsive actuators: (a) actuators with different magnetic domains using hard magnetic particles, (b) actuators with complex magnetic structures using soft magnetic particles, and (c) actuators with particle chains using mold casting and soft magnetic particles.

Some interesting magnetic actuators capable of executing complex 3D movements have been achieved by using particles of magnetically hard materials (Figure 1a). For example, Kim et al.^42^ used a silicone-based composite ink containing neodymium–iron-boron (NdFeB) microparticles to make 3D-printed materials while applying a magnetic field to orient the particles. Xu et al.^43^ used permanent magnetized microparticles of NdFeB alloy for creating regions of arbitrary magnetization direction in planar soft elastomers. These planar actuators demonstrated complex shape changes as well as locomotion, grasping, and crawling in response to applied magnetic fields. This strategy has also been used to develop magneto-responsive origami soft actuators^44,45^ and magneto-responsive soft actuators for specific applications.^46−48^ All of these magnetic actuators rely on the high remanent magnetization of the magnetic material. Unfortunately, these approaches do not meet the biocompatibility and biodegradability requirements of biomedical applications.

A second approach to magnetically controlled actuation is to create regions of anisotropic magnetic susceptibility rather than regions of magnetic remanence. Such regions have negligible magnetization in the absence of a magnetic field. These regions can be formed by applying a magnetic field during the cure of the pregel, which will cause the particles to form chain-like structures that are aligned with the field. These particle chains form to reduce the magnetostatic contribution to the free energy of the system, which, in turn, increases the magnetic susceptibility. Such regions will tend to align with the direction of the subsequent actuating field in the fully cured material, thus imbuing the composite with shape memory (Figure 1b). Because magnetic remanence is not required, nontoxic and magnetically soft iron and iron oxide magnetic nanoparticles (MNPs) can be employed. However, this method would seem to be a challenge for achieving complex actuation deformations, since the formation of more complex magnetic regions than those in refs (20) and (49) would require complex magnetic-field geometries, which might be difficult to engineer. In fact, Kim et al.^49^ and Goudu et al.^20^ have fabricated magnetic actuators consisting of regions having MNP chains oriented in different directions. The resulting planar-like actuators responded by aligning the MNP structures of each region in the direction of the applied field, giving rise to different deformations in different regions.

In the current work, we present an alternative approach to clustering magnetic particles in order to generate shape-memory materials that actuate in 3D. Instead of trying to create complex magnetic-field geometries that can be applied to the curing pregel, we simply apply a spatially uniform magnetic field to a pregel that is in a mold of appropriate shape for the composite in its desired fully field-actuated geometry (Figure 1c). The particle chains that form along the field lines become permanently fixed at the gel point, after which the field can be removed and gelation completed with the composite in the desired off-field geometry. The final magnetic shape-memory material returns to the shape of the original mold by applying a strong, uniform magnetic field and can continuously morph into this shape by applying fields of intermediate strength. In this paper, we demonstrate this approach by creating both bending and twisting actuators and a biomimetic structure that imitates the motion of butterfly wings. We emphasize that this mold-casting approach requires neither permanently magnetized particles nor complex magnetic-field geometries, which is a significant simplification compared to current methods. Furthermore, the use of nontoxic iron particles in combination with natural alginate biopolymers makes these shape-memory magnetic hydrogels suitable as biocompatible soft magnetic actuators.

In the following, we analyze the microstructural changes that take place under a magnetic field in alginate-based magnetic hydrogels. We then demonstrate that the alginate-based magnetic hydrogels can be used to fabricate unconstrained actuators based on the approach of the complex magnetic-field geometries of refs (20) and (49). The mold-casting approach is then demonstrated, and a theoretical model for some of the novel actuators developed in this work is described.

## Results and Discussion

### Microstructural Changes under a Magnetic Field

**Figure 2:**
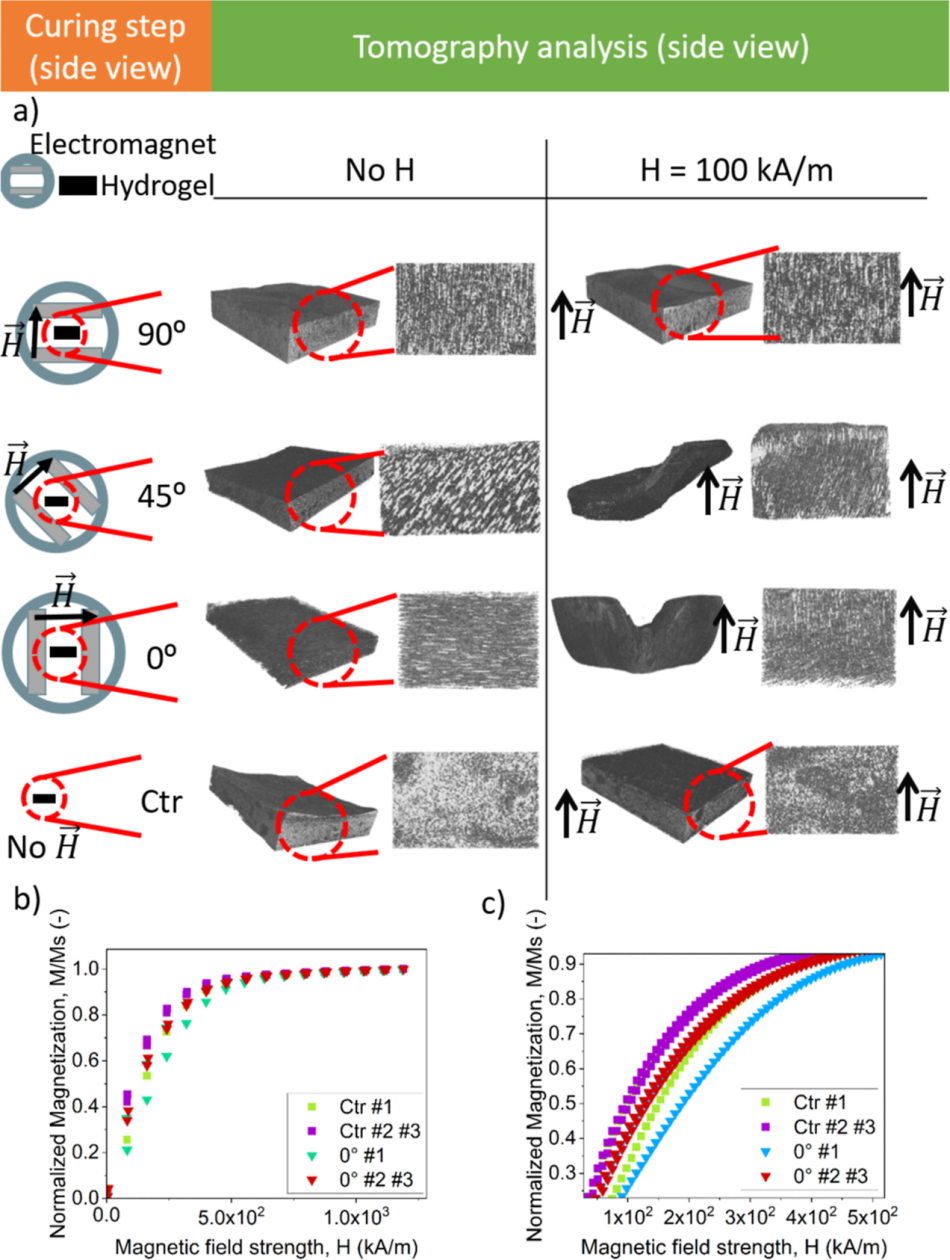
X-ray tomography images and magnetization curves of disk-shaped magnetic hydrogels cured under a unidirectional homogeneous magnetic field (55 kA/m) applied at different angles (0, 45, and 90°, indicated in the figure) with respect to the main plane of the hydrogel samples. Results for a magnetic hydrogel cured in the absence of a magnetic field (Ctr sample) are also shown. (a) X-ray tomography. Images for samples in the absence of a magnetic field and under the application of a magnetic field perpendicular to the main plane of the hydrogel samples are shown. In all cases, a general perspective of the sample, accompanied by a zoomed lateral view, are shown. All samples had a particle concentration volume of 1%. The direction of the applied magnetic field, *H*, is indicated by arrows. (b,c) Normalized magnetization curves of samples containing a particle volume concentration of 5%.

The microscopic structural changes of the magnetic hydrogels under an actuating magnetic field were visualized by X-ray microcomputed tomography (microCT) assays. These assays revealed that when a magnetic field was applied during gelation (curing step), the magnetic particles aggregated into chain-like structures aligned with the field direction (0°, 45°, or 90° with respect to the main plane of the hydrogel), which were rendered permanent by gelation (Figure 2a). As a control (Ctr) sample, we analyzed alginate hydrogels with an isotropic distribution of magnetic particles (no field applied during gelation). When a magnetic field (100 kA/m) was subsequently applied in the direction perpendicular to the main plane of the hydrogel, no microstructural changes in the particle distribution were observed for the sample gelled in the absence of a magnetic field (Ctr sample) (Figure 2a). This indicates that the particles embedded in the polymer network were not able to migrate under a magnetic field of 100 kA/m because the elastic forces of the polymer network dominate the magnetostatic forces between particles. Similarly, no microstructural changes were observed in the hydrogel with the particles aggregated into chains aligned at 90°, i.e., parallel to the actuating field lines, since they are already at the minimum energy orientation. However, samples with particle structures aligned at 0 and 45° demonstrated reorientation at the microscopic level (Figure 2a) because of the tendency of the particle chains to align with the actuating magnetic field, which minimizes their magnetostatic energy. This rotation of the particle chains results in bending of the magnetic hydrogel. Moreover, by controlling the angle of the particle structures with respect to the plane of the hydrogel, it is possible to control the resulting bending angle of the hydrogel under a perpendicularly applied magnetic field.

In order to further investigate the possibility of field-induced particle migration in magnetic hydrogels, we subjected pristine samples to three cycles of saturating magnetic fields (Figure 2b,c). The first cycle gave smaller values of magnetization at low and medium values of the applied magnetic field than the successive cycles, for which there were no subsequent changes. These measurements indicate particle migration in the initial cycle of magnetization in such a way to reduce their magnetic energy, thus increasing the composite susceptibility. Particle migration in these magnetization experiments (Figure 2b,c) does not contradict its absence in the actuation experiments (Figure 2a), since the applied magnetic field was 1 order of magnitude higher in the former (1000 kA/m) than in the latter (100 kA/m). Indeed, in a previous study, the migration of magnetic particles was demonstrated in similar magnetic alginate hydrogels under a magnetic field of 280 kA/m.^50^ Note also that differences in magnetization between the first cycle and the successive cycles were the largest for the Ctr sample, which due to its originally isotropic distribution of particles was far from the configuration of a minimum of energy.

### Planar Actuators Cured in the Off-State Configuration (Horizontal Position)

**Figure 3:**
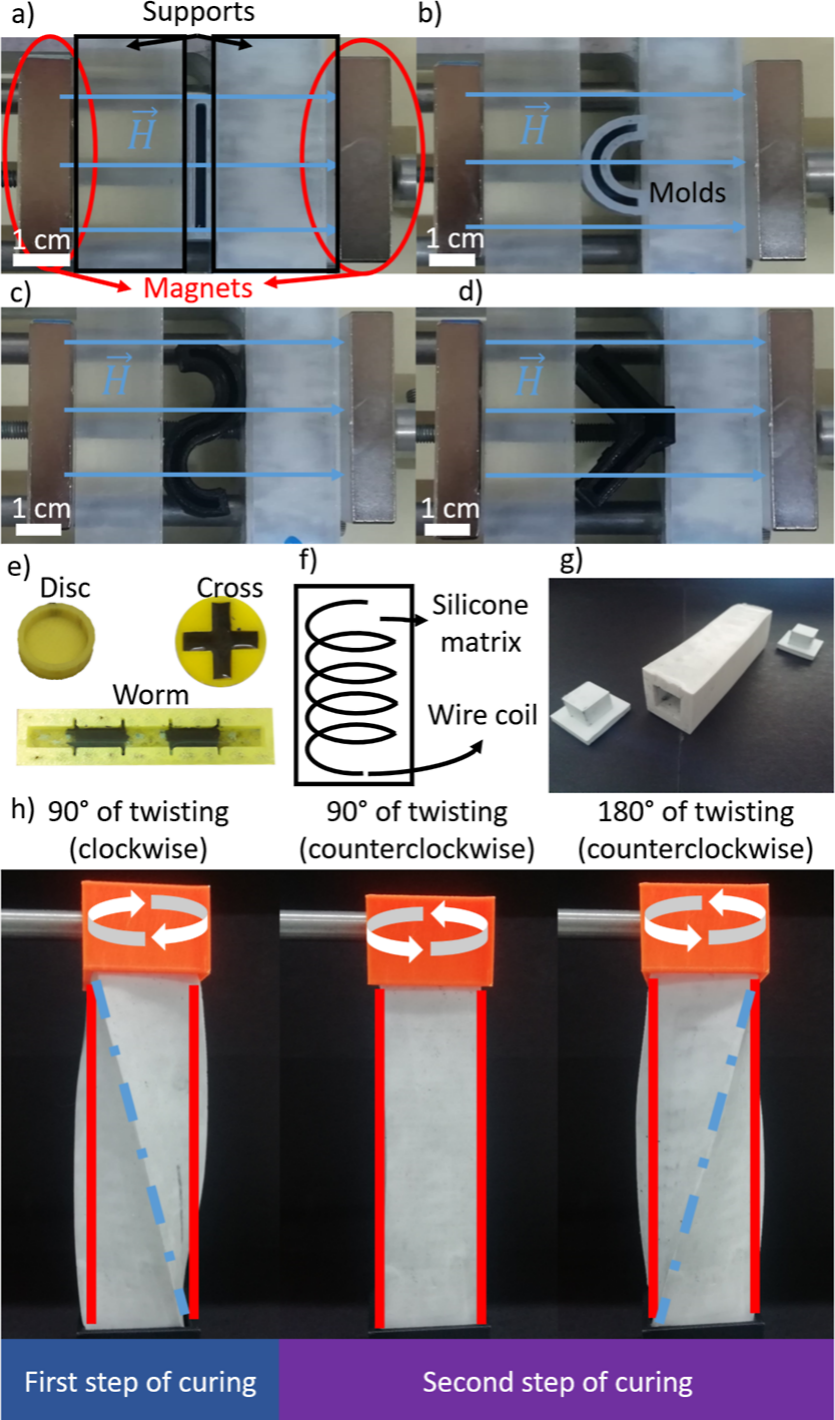
(a–e) Photographs of different PLA molds used as containers for hydrogel cross-linking. (a) Rectangular mold, (b) semicircular mold, (c) S-shaped mold, (d) buttery mold, and (e) disc-, cross-, and worm-like molds. The supports as well as the magnets used for application of magnetic fields during cross-linking are also shown in photographs (a–d) —magnetic field direction is indicated by arrows. (f) Scheme of the silicone mold incorporating a wire coil in the lateral walls. (g) Photograph of the final silicone mold together with the corresponding plugs. (h) Pictures of the silicone mold twisted at different angles (indicated).

**Figure 4:**
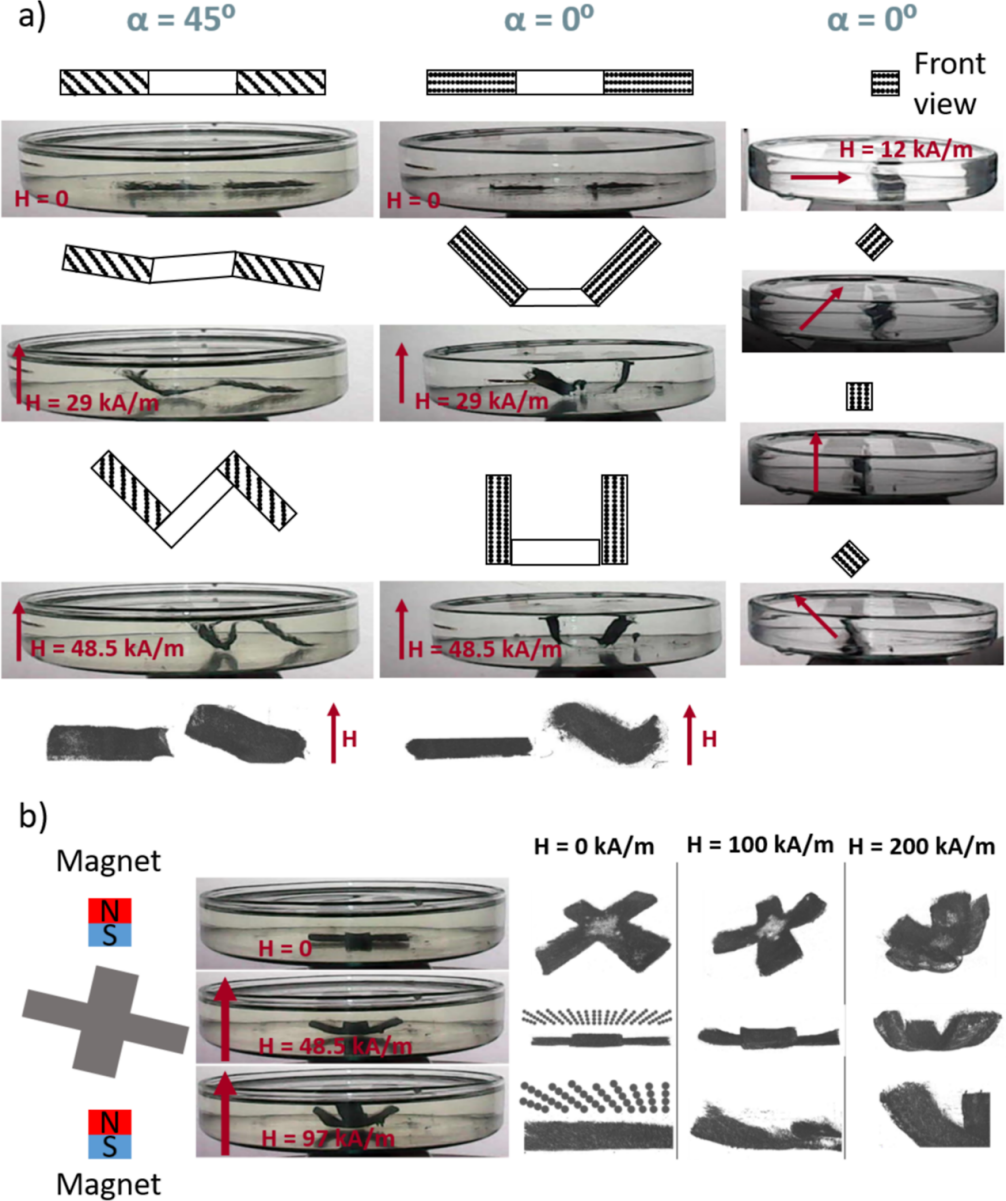
Actuation behavior of planar actuators cured in the off-state configuration. (a) Planar actuators consisting of three alternating magnetic/nonmagnetic/magnetic bands, with magnetic particles aligned at different angles (α, indicated) with respect to the actuator plane in the off-state (*H* = 0). The magnetic field strength, *H*, and its direction during actuation are indicated, and photographs are accompanied by sketches that illustrate the three bands of the actuators and the particle chains. The bottom row shows images of microCT under a magnetic field. (b) Cross-shaped actuator with particle chains progressively deflected from the vertical direction. A sketch of the configuration used during curing is shown in the left column, whereas the three columns to the right show microCT images of the whole actuator (top and middle rows) and a detail of one arm (bottom row). Sketches of the orientation of particle (represented by black dots) chains in the off-state (*H* = 0) are also provided.

As a simple demonstration of the tendency of particle chains to align in the direction of the applied magnetic field, we fabricated planar magnetic hydrogels in a magnetic field oriented at an angle smaller than 90° with respect to the plane of the hydrogel (i.e., the horizontal direction). We tested various patterns (Figure 3e) and directions of the structuring magnetic field to create a variety of actuators (Figure 4). For example, under a vertical actuating magnetic field, planar hydrogels consisting of three alternating magnetic/nonmagnetic/magnetic bands mimic the bending of worms when crawling, if the direction of the structuring magnetic field was at an angle of 45° from the horizontal axis (left column of Figure 4a). On the other hand, if the structuring magnetic field had been directed along the main, horizontal axis of the worm-like actuator (0°, longitudinal direction), then in a vertical actuating field the composite forms the letter U (central column of Figure 4a). Finally, for a similar three-band actuator cured in a structuring field directed along its transverse axis, a simple rotation was observed under a magnetic field (right column of Figure 4a). In all of these cases, the actuation is a simple consequence of the tendency of chains to align with the actuating magnetic field.

A four-arm gripper with a more progressive bending behavior was made by curing a cross-shaped magnetic hydrogel under the nonhomogeneous magnetic field created by two opposing permanent magnets of smaller size than the cross-shaped actuator placed with the line connecting their centers coincident with the symmetry axis of the cross-shaped actuator.^20^ This inhomogeneous magnetic field induced particle chains to progressively deflect from the vertical direction, starting from the midpoint, where they were parallel to the vertical direction. We reproduced this actuator (Figure 3e) in order to analyze the bending behavior in relation to changes in the microstructure studied by microCT (Figure 4b). As is seen, the bending behavior in the on-state is a result of progressive alignment with the applied magnetic field of the particle chains, which thus results in circular bending.

All of these planar actuators showed complete reversibility, returning to their initial off-state when the magnetic field was removed, and they did not show an appreciable decrease in their response when subjected to successive on/off cycles. Furthermore, their actuation response was rapid, taking place in all cases in less than 3.64 s after application or removal of the magnetic field. It should be noted that by means of a suitable combination of hydrogel geometry and arrangement of magnetic particles during cure, planar actuators with virtually any complex movement can be achieved as a simple consequence of the tendency of magnetic particle chains to align with the actuating magnetic field.

### Actuators Cured in the On-State Configuration

**Figure 5:**
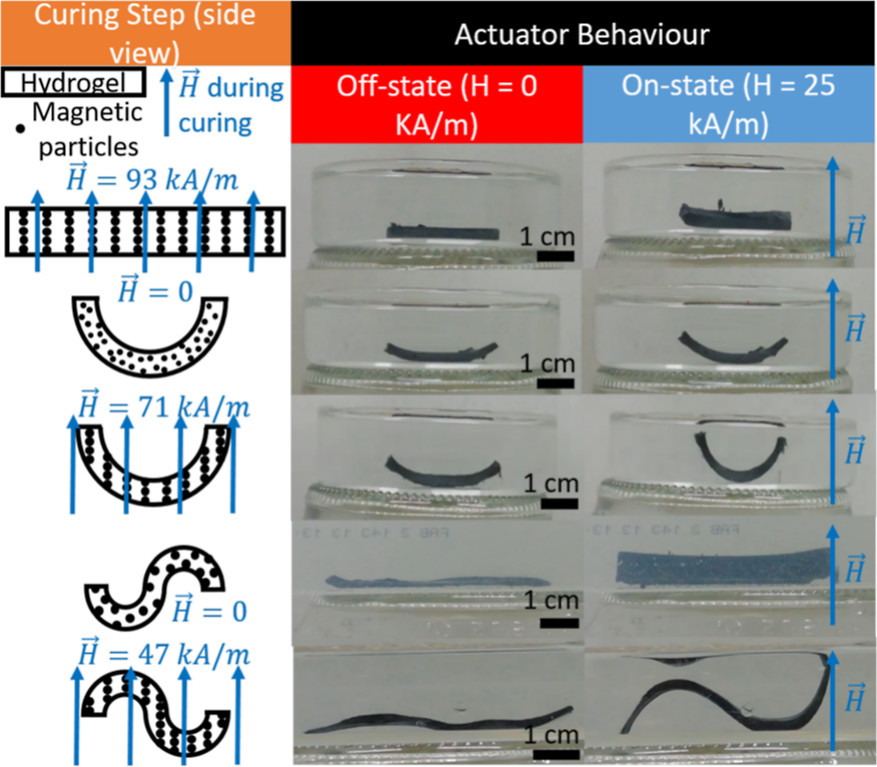
Actuation behavior of planar actuators cured in the on-state configuration. The left column presents sketches of the curing step, with indication of the shape kept during curing, the presence or absence of a vertical magnetic field, and sketches of the expected distributions of magnetic particles (Fe-CC, represented by black dots). The central column and the right column, respectively, show photographs of the magnetic hydrogels in the absence of an applied magnetic field (off-state) and in the presence of a vertically applied magnetic field (on-state). Each row corresponds to a different hydrogel cured under different conditions, as indicated in the left column.

Creating planar actuators with complex deformations by the approach discussed in the previous subsection could require complex magnetic fields that might be difficult to achieve. In this subsection, we explore the simpler approach of curing the magnetic hydrogels in a mold of the desired on-field shape in a uniform, vertical magnetic field (Figure 3), to create magnetic shape-memory materials. We first demonstrate this approach by field-curing a semicircular shape and an S-like shape (Figure 5). As control samples, we cured magnetic hydrogels in these molds in the absence of a magnetic field as well as a magnetic hydrogel cured in the shape of a parallelepiped under a vertical field (Figure 5). Because of the low elastic modulus of these field-structured hydrogels, they flattened after being extracted from the molds (Figure 5, off-state). But in a vertical actuating magnetic field, they immediately returned to their field-cured shape (Figure 5, on-state). Indeed, the curvature (defined as the inverse of the radius) was 9 × 10^–5^ μm^–1^ for the semicircular actuator in the on-state vs 10^–4^ μm^–1^ for the mold in which it was cured. Of course, the sample field-cured in the shape of a parallelepiped did not bend in the on-state nor did the sample cured in an S-like shape in the absence of an applied field.

Two additional shape-memory magnetic hydrogels were field-cured in semicircular molds: one convex and the other concave (Supporting Information Figure S1). In the off-field state, these relaxed to nearly a flat shape but quickly recovered their field-cured shape in an applied field. A control sample that was field-cured in a planar geometry did not experience any significant actuation, though it is likely that some small level of magnetostriction occurred.

**Figure 6:**
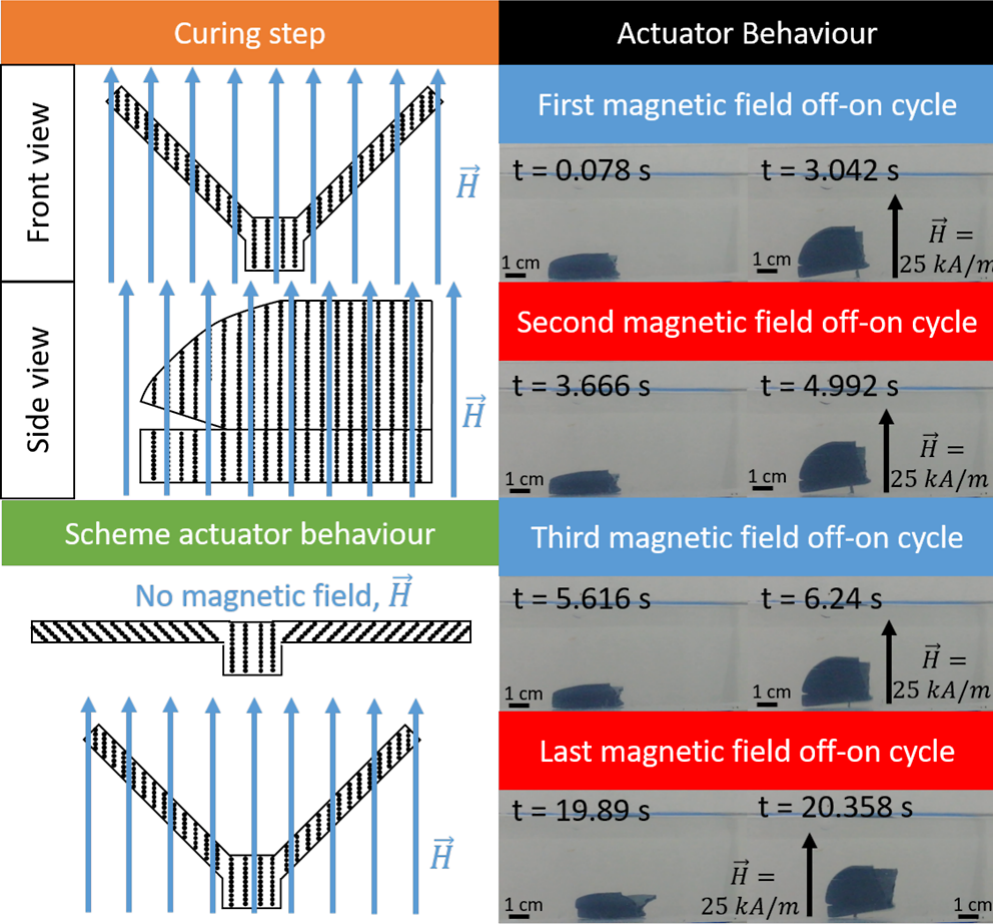
Actuation behavior of a planar actuator imitating a butterfly. The left column presents sketches of the curing step and actuation behavior; note that the expected distributions of the magnetic particles (represented by black dots) are illustrated, as well as the direction of the magnetic field. The right column presents photographs of the butterfly for different cycles of application (on-state) and removal (off-state) of the magnetic field.

Finally, we explored the possibility of creating biomimetic dynamics^51^ by fabricating a butterfly (Figure 6). To achieve a fluttering motion of the wings, we cured the magnetic hydrogel in a vertical magnetic field, with the wings at a 45° angle to the field. After curing, we submerged the butterfly in water and noted that in the absence of an applied magnetic field the butterfly wings were approximately horizontal. When a vertical magnetic field was applied, the wings quickly returned to their on-field position, which resulted in a hop. Successive field cycles resulted in locomotion of the butterfly (Figure 6, Supporting Information Video S1 and Supporting Information Figure S2). The response of the butterfly to the magnetic field was almost instantaneous, requiring 0.304 ± 0.003 s to raise the wings and 0.224 ± 0.003 s to lower them.

### Twisting Actuators

At this point, the magnetic shape-memory materials we have fabricated lack chirality, so torsional actuation of an unconstrained material is not possible. In this section, we demonstrate the fabrication of chiral shape-memory materials that exhibit twisting in an applied field. These composites are fabricated by a somewhat different process than that described above: instead of fully field-curing in the desired, twisted actuation state, they are only cured to the gel point in this state, at which time the field is turned off and they are then fully cured in the untwisted off-field state. Because the elastic modulus of the hydrogel at the gel point is zero, or at least negligible in practice, the final material has no tendency to relax to the on-field geometry upon removal from the mold, and gravity plays no role in achieving the off-field conformation. Fischer and Menzel have reported a theoretical model analyzing the effect of a similar two-step preparation protocol on the particle arrangement of dispersions of magnetic particles in a polymer network and their response to magnetic fields after curing.^17,19^

**Figure 7:**
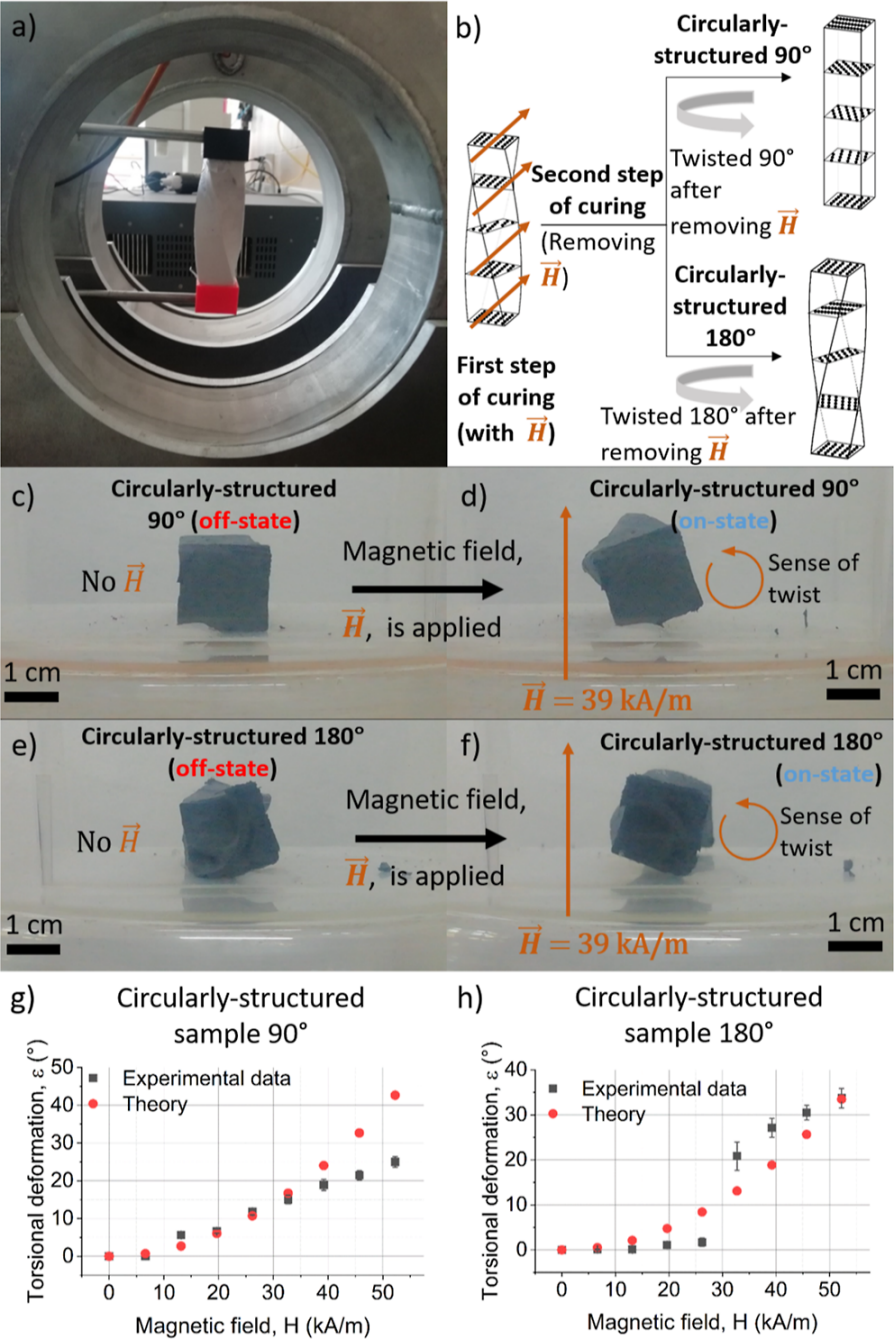
(a) Experimental setup used to obtain the circularly structured magnetic composites, where the magnetic field generated by the coils was perpendicular to the main symmetry axis of the parallelepiped sample. (b) Until the gel point, curing was carried out with the sample twisted 90° (clockwise) and under the applied magnetic field; after the gel point, the field was removed and the sample was twisted 90° or 180° (counterclockwise) and maintained until complete curing, note that the expected distributions of the magnetic particles (represented by black dots) are illustrated, as well as the direction of the magnetic field. (c,d) Illustration of the actuation behavior of the circularly structured sample twisted 90° during the second step of curing. (e,f) Illustration of the actuation behavior of the circularly structured sample twisted 180° during the second step of curing. (g,h) Torsional deformation as a function of applied magnetic field for circularly structured samples.

We prepared the chiral magnetic hydrogels in flexible parallelepiped silicone molds (Figure 3) that could be twisted during cure. We prepared four different hydrogels that differed in the direction of the structuring field (either parallel or perpendicular to the long axis of the mold) and the angle at which the molds were twisted after the removal of the magnetic field (Table 1). We refer to composites structured with the field normal to the long axis of the mold as circularly structured and those structured with the field parallel to this axis as helically structured. The twist angle refers to the final angular position of the mold relative to that during the cure to the gel point (Figure 3h). During field cure, the molds were held vertically, with the upper end twisted 90° clockwise (Figure 7a). At the gel point, the magnetic field was turned off, and the upper end of the molds were twisted 90° or 180° counterclockwise, obtaining in this way four different configurations of particle aggregates; see Supporting Information Videos S2, S3, S4, and S5. Note that because of the nonlinear distribution of stress under simple torsion in samples having a square section,^52^ in the off-state the particle structures are more complicated than linear chains. However, to simplify the analysis, we can approximate these structures as linear chains (Figure 7b).

**Table 1:** Identification of the Magnetic Hydrogels Prepared in This Work

			prepared in silicone mold	
sample	particle concentration (% v/v)	prepared in PLA mold (addition of CaCl_2_)	orientation between the magnetic field and the longitudinal axis	angle of twisting (counterclockwise) after removing the magnetic field θ_*m*_
microCT analysis	1	yes		
disc, cross, worm, rectangular, semicircular, S-shape, butterfly	5	yes		
circularly structured sample 90°	15		⊥	90°
circularly structured sample 180°	15		⊥	180°
helically structured sample 90°	15		∥	90°
helically structured sample 180°	15		∥	180°

First, we discuss the results for the circularly structured magnetic hydrogels (Figure 7a). In the absence of an applied magnetic field, the hydrogel that was twisted θ_*m*_ = 90° during full cure exhibited no residual twist (Figure 7c), whereas the 180° sample had a slight residual twist, probably because it had field-cured slightly beyond the gel point (Figure 7e). Under a magnetic field applied perpendicular to their main axis, both actuators responded by torsional movement due to the tendency of the particle structures to align with the magnetic field (Figure 7d,f). The torsional response to the magnetic field was very fast in all cases (Supporting Information Video S6) above a threshold magnetic field strength (*H*), and the angle of torsion with respect to the absence of applied magnetic field (off-state) increased progressively with *H* (Figure 7g,h). For the magnetic hydrogels twisted 90° during the second step of curing, a maximum experimental twisting angle of 25° ± 1° was obtained at *H* = 52 kA/m. For the samples twisted 180° during the second step of curing, a higher maximum deformation, 33.7° ± 2° at *H* = 52 kA/m, was obtained. Furthermore, in Figure 7h, it can be observed that a minimum threshold value of the applied magnetic field of *H* = 32.7 kA/m was required in this latter case to obtain an appreciable response.

**Figure 8:**
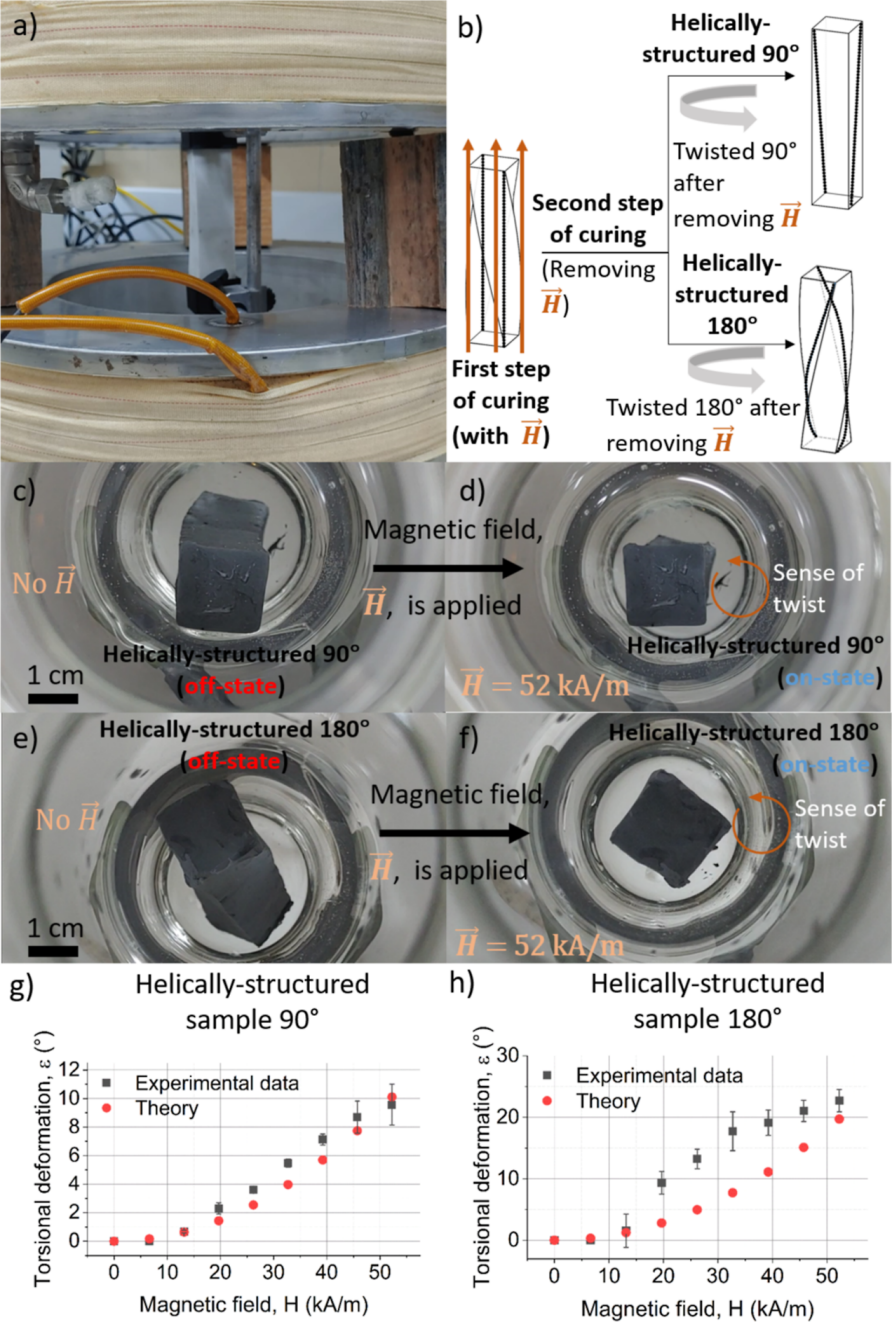
(a) Experimental setup used to obtain the helically structured magnetic composites, where the magnetic field generated by the coils was parallel to the main symmetry axis of the parallelepiped sample. (b) Until the gel point, curing was carried out with the sample twisted 90° (clockwise) and under the applied magnetic field; after the gel point, the field was removed and the sample was twisted 90° or 180° (counterclockwise) and maintained until complete curing, note that the expected distributions of the magnetic particles (represented by black dots) are illustrated, as well as the direction of the magnetic field. (c,d) Illustration of the actuation behavior of the helically structured sample twisted 90° during the second step of curing. (e,f) Illustration of the actuation behavior of the helically structured sample twisted 180° during the second step of curing when an external magnetic field was applied parallel to the symmetry axis of the parallelepiped sample. (g,h) Torsional deformation as a function of applied magnetic field for helically structured samples.

We next investigated the actuation behavior of the helically structured magnetic samples (Figure 8a). Similarly to the circularly structured samples, in the absence of an applied magnetic field, the hydrogel that was twisted 90° during full cure had no twist (Figure 8c), whereas the 180° sample had a slight residual twist (Figure 8e). Above a minimum applied magnetic field, both samples progressively twisted back to their field-cured shape (Figure 8g,h). Maximum angles of twisting with respect to the off-state for helically structured magnetic hydrogels were 9.6 ± 1.4° and 23 ± 2° (at *H* = 52 kA/m) for the 90° and 180° materials, respectively. Similar to the circularly structured hydrogels, the maximum twisting achieved for helically structured magnetic hydrogels was higher for the 180° than the 90° samples. The twisting of the helically structured samples was substantially smaller than for the circularly structured samples.

It should be noted that we did not achieve twisting actuation for magnetic hydrogels fully cured in the applied field, i.e., using the approach in the subsection Actuators Cured in the On-State Configuration.

### Fluidic Application, Reversibility, and Stability of Magnetic Hydrogel Actuators

The actuation behavior of magnetic hydrogels can be used for different applications. For example, in a previous work, we reported a valve remotely actuated by a magnetic field that was based on the dimensional changes of magnetic hydrogels under a magnetic field.^8^ More common magnetic hydrogel actuators are used as grippers for cargo grabbing, transportation, and release by controlling magnetic field inputs.^20,21,43^ Here, we report a novel fluidic application based on torsional actuation behavior. The fluidic device consisted of a circularly structured twisting actuator (hydrogel sample twisted 180° during the second step of curing) vertically placed and fixed to the floor by its bottom surface, otherwise free to rotate under the action of an applied magnetic field. A rigid plastic channel was fixed to the upper surface of the hydrogel by gluing (Supporting Information Figure S3). An orange-tinted solution of water flows through this channel. Actuation was achieved by application of a vertical magnetic field with a coaxial pair of Helmholtz coils. By modifying the intensity of the applied magnetic field, the flow direction could be remotely modified, allowing the delivery of the water solution to different receiving channels (Supporting Information Video S7). This proof-of-concept application might be used, for example, to construct an automatic station for delivery of quantified solutions for controlled reactions or to promote different physicochemical processes such as crystallization.

An important question arising at this point is on the reversibility of shape deformation, which is essential for applications of actuators. As any material, magnetic hydrogels of the present work must have a deformation limit, yield point, above which a residual deformation remains.^53^ To investigate this, we subjected our torsional actuators to torsional deformation of stepwise increasing angle, releasing the stress after each step (Supporting Information Video S8). We found that even for an angle of 40°, the actuators still behaved as an elastic material, with negligible residual deformation remaining after removal of the stress (Supporting Information Figure S4). Since in all cases the twisting angles obtained by magnetic actuation are considerably smaller than 40°, for operation conditions like these of the present work, the lack of reversibility of the magnetic hydrogel actuators does not constitute a problem.

We finally investigated the stability of the actuators under working conditions by subjecting them to 120 cycles of application and removal of the magnetic field, for a total duration of 20 min. This experiment was carried out for butterfly-shaped and circularly structured 180° actuators (Supporting Information Video S9 and Supporting Information Figure S5). As observed, actuators maintained their responsiveness to the application/removal of the field after this large number of working cycles, although in the case of the butterfly-shaped actuator the response became less intense over time, due to an increasing residual angle in the off-state. Note, however, that this residual angle decreased over time once the actuation experiments were completed, going from 28° immediately after the last working cycle to 15° after 40 min, supporting the negligible value of the permanent residual deformation of the hydrogels of the present work under the actuation deformation they experienced; note that the deformation angle of the pristine butterfly actuators was 8°.

## Theoretical Model

Here, we investigate theoretically both circularly and helically structured torsional actuators. For simplicity, we modeled the parallelepiped samples as cylinders.

### Modeling Actuation of the Circularly Structured Magnetic Composite

**Figure 9:**
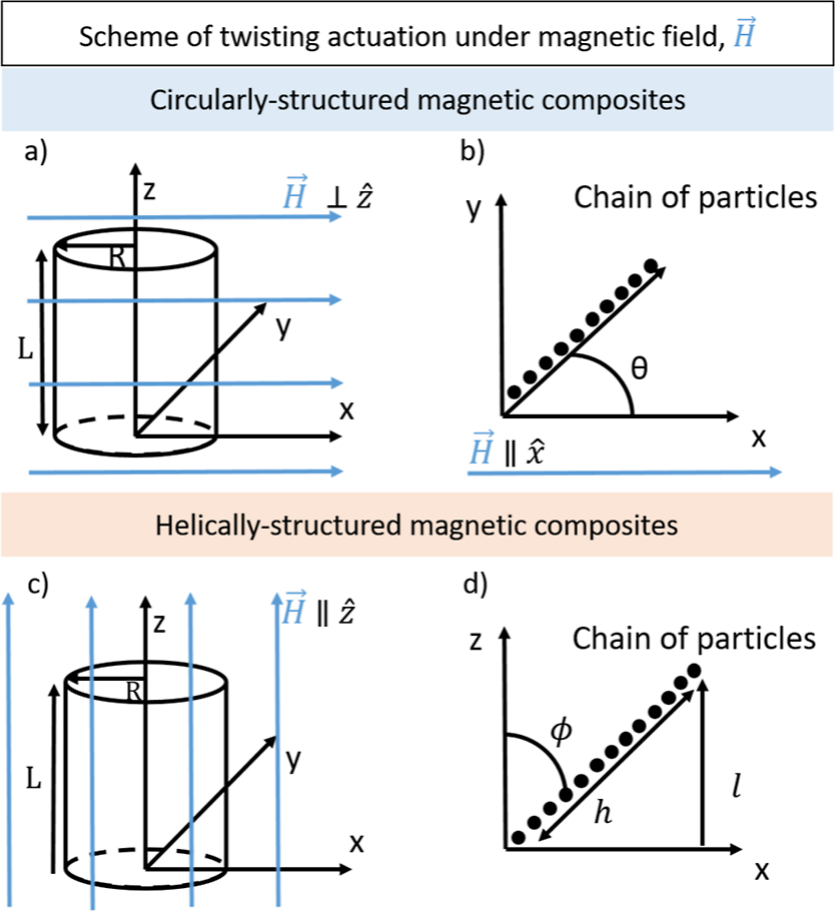
Scheme of the samples in the circularly structured magnetic composite model: (a) under an applied magnetic field and (b) angle of the chains of particles with the magnetic field. Scheme of the samples in the helically structured magnetic composite model: (c) under an applied magnetic field and (d) angle of the chains of particles with the magnetic field.

We consider the case where magnetic particle chains are formed normal to the cylindrical axis of a composite and make an angle θ to the *x* axis. Such a composite is created by applying a uniaxial magnetic field perpendicular to the axis of cylindrical symmetry to a magnetic particle suspension in a polymeric solution until the gel point is reached (Figure 9a,b), upon which time the field is removed, and the composite is twisted by an angle θ_*m*_ and allowed to fully cure. Two questions arise. First, what is the preferred orientation θ of this composite in the presence of an orthogonal field, and second, how much will the angle θ_*m*_ change in such a field. The latter change we refer to as its actuation, i.e., torsional deformation ε (Figure 7). For this, we need the magnetostatic energy as a function of the composite angle θ to compute its equilibrium (minimum) value, *U*, which can be written as (see Supporting Information Section S1)
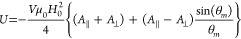
1Here, *V* is the volume of the composite, μ_0_ is the permeability of vacuum, *H*_0_ is the applied magnetic field, and  and , with χ_∥_ and χ_⊥_ being the composite susceptibilities parallel and perpendicular to the field-structuring direction and *n* the demagnetization factor for a field applied normal to a cylinder, which only depends on the aspect ratio of the sample and the susceptibility of the material.^54^ As a special case, when θ_*m*_ = 180°, the energy is independent of the orientation and the difference *A*_∥_ – *A*_⊥_.

Having established the orientation of the composite in a magnetic field, we can now examine its actuation, which we define as the change in the angle θ_*m*_ in the presence of a transverse magnetic field. This actuation can be measured with one end of the composite held in a fixed orientation, or we can assume that the composite is free to rotate to its equilibrium position, which can result in a more subtle change. In the following, we consider the latter case. When the cylindrical composite at its equilibrium orientation is subjected to a field normal to its cylindrical axis, the angle θ_*m*_ will decrease to θ_*m*_ – ε. The first order of energy change Δ*U*(ε) ≡ *U*(θ_*m*_ + ε) – *U*(θ_*m*_) is
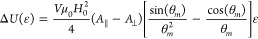
2where we have used a Taylor expansion for the trigonometric function. Because the trigonometric function in this expression is positive for the region of interest here, ε is negative, as expected. For θ_*m*_ ≤ π/2, this trigonometric term is approximately .

The twisting energy of a cylinder is , where *G* is the shear modulus of the composite of radius *R* and length *L*. Adding this energy to the magnetostatic energy and minimizing the energy give an expression for the angular actuation.
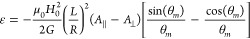
3

The actuation is quadratic in the aspect ratio of the cylinder and the field, inverse in the elastic modulus, and independent of the overall scale of the cylinder. The effective magnetic susceptibility anisotropy term is critical in determining the magnitude of the actuation. The magnetic susceptibility of composites comprised iron particles structured into chains by a uniaxial field has been reported, with care taken to accurately compute the internal field of the solid rectangular samples.^55^ The results in Supporting Information Figure S6a show that the susceptibility χ_∥_ along the direction of the applied structuring field increases linearly with the particle volume fraction and that the susceptibility χ_⊥_ is significantly lower in the perpendicular direction.

Unfortunately, the effective susceptibilities parallel and perpendicular to the structuring field, *A*_∥_ and *A*_⊥_, are greatly reduced due to the strong demagnetizing fields for a field applied normal to the cylindrical axis. Supporting Information Figure S6b shows these effective susceptibilities: their difference is a maximum at around 15 vol % particles, where it is roughly 0.4.

It is interesting to compute the predicted actuation for a 15 vol % cylinder with an aspect ratio of 20, e.g., 1 cm diameter and 10 cm long. For an initial value of θ_*m*_ = 90°, the trigonometric function in the actuation expression is about 0.41. The difference between the effective susceptibilities is 0.40, giving . For a field of 200 Oe (1.6 × 10^4^ A/m), the magnetic energy density is 320 J/m^3^. The shear modulus of a hydrogel is on the order of 10^5^ Pa = 10^5^ J/m^3^, giving ε ≅ 6°. Using magnetic flakes would greatly increase the composite susceptibility in the direction of the structuring field and greatly decrease the perpendicular susceptibility, but in the most extreme case imaginable the effective susceptibility contrast cannot exceed *A*_∥_ – *A*_⊥_ = 2, since it is the inverse demagnetization factor of a cylinder normal to its axis. This change would increase the estimated actuation by a factor of 5.

A comparison between the experimental results for a 15 vol % cylinder with 17 mm in diameter and 81 mm in length can be seen in Figure 7, wherein we compare the torsional deformation as a function of the magnetic field for two different values of θ_*m*_, 90° and 180° (Figure 7g,h). For θ_*m*_ = 90° (circularly structured sample prepared at 90° during the second step of curing), we can observe that the model fits the experimental results until a magnetic field strength of 33 kA/m is reached. After this value of *H*, the experimental data increase more slowly than the theoretical data, with the experimental data achieving a maximum value of 25 ± 1° and the theoretical data achieving a maximum value of 43°. Whereas, for θ_*m*_ = 180° (circularly structured sample prepared at 180° during the second step of curing), a larger difference is observed at a medium field between the theoretical and experimental results, which has a large increase of the torsional deformation for *H* = 32 kA/m, but after this a better agreement between experiment and theory is obtained.

### Modeling Actuation of the Helically Structured Magnetic Composite

To create a helically structured magnetic composite, a cylindrical mold containing a gelling liquid dispersion of magnetic particles is first twisted, and then a field is applied parallel to its cylindrical axis. Shortly after the gel point is reached, the field is removed and the mold is untwisted, resulting in particle chains that spiral up the cylinder, Figure 9c,d. When a field is applied to the final composite, again along the cylindrical axis, the chains will attempt to align with the field, causing twisting actuation of the composite.

The chains at the surface of the cylinder of radius *R* will make an angle ϕ_*m*_ relative to the cylindrical axis, and this angle is related to the pitch of the composite, which is the length over which the composite twists a full turn. In one turn around the cylinder, the surface chains rise a distance *l*. The length of these chains will thus be . The angle ϕ_*m*_ is given by  and the pitch by *l* = 2π*R*/tan(ϕ_*m*_). For a composite of length *L* that was fabricated with a pregel mold twist of θ radians *l* = 2π*L*/θ, the relation between the two angles is . For example, for a 10 cm long composite made with a 90° pregel twist, the surface chains make an angle of ∼8.9° relative to the cylindrical axis. The chains in the interior of the cylinder make a progressively smaller angle that vanishes at the center of the cylinder.

In this case (helically structured composites), the change in the magnetic energy of the composite upon torsional deformation is given by (see Supporting Information Section S2)
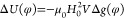
4where
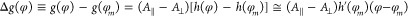
5with *h*(ϕ) and its derivative *h*′(ϕ) being functions of ϕ, whose expressions are given by Supporting Information eqs S12 and S14.

The elastic twisting energy of the cylinder is given by
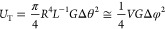
6where *G* is the shear modulus, Δθ = θ – θ_*m*_, and Δϕ = ϕ – ϕ_*m*_ is the displacement in terms of the chain angles. Minimizing the sum of elastic and magnetic energies gives the expected torsional actuation in an applied field.
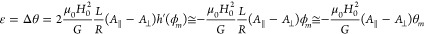
7

High-aspect-ratio composites will give the greatest effect as these will minimize the effect of demagnetizing fields. Note that because θ_*m*_ = 2π*L*/*l*, the actuation is proportional to the length at a constant pitch and inversely proportional to the pitch at a constant length.

In the case where the cylinder is long enough that the demagnetizing fields are negligible, then we can use the experimental expressions for the susceptibilities of uniaxially structured composites made of carbonyl iron particles^55^ to give the dependence on the volume fraction
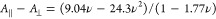
8where ν is the volume fraction, with ν ≤ 0.3. This function is essentially a maximum (constant value) over the volume fraction range of 0.15–0.25, (see Supporting Information Figure S6).

Using the theoretical model developed in this subsection, we were able to calculate the theoretical torsional deformation for these samples as a function of the magnetic field, and as in the subsection, we compared them with the experimental results (Figure 8). The samples had a length of 81 mm and a diameter of 8.5 mm, and for helically structured samples twisted 90° during the second step of curing, the model predicts the experimental data, although the theoretical data increase with *H*^2^ and the experimental data follow a linear trend with *H* (Figure 8g). As for the helically structured sample twisted 180° during the second step of curing, the experimental data show larger differences with the theoretical model compared with the other three types of samples. These results clearly show a different trend between the two curves, where the experimental data follow a saturation trend, while the theoretical data follow a quadratic trend with the magnetic field. Despite the difference between the trends in the curves, the experimental and theoretical data reach a close value of the maximum torsional deformation (Figure 8h).

## Conclusions

We prepared a variety of magnetic shape-memory materials by creating uniaxial magnetic susceptibility anisotropy in alginate gels containing iron carbonyl particles. These gels were partially or fully cured in a uniform uniaxial magnetic field applied to the pregel while confined to a mold of the desired geometry in order to imbue the material with a permanent shape memory. Due to gravitational distortion of the field-cured gel or to remove the field at the gel point and fully curing the material in another conformation, one can create a material of arbitrary shape that will quickly, continuously, and reproducibly return to its memory shape when subjected to a uniform magnetic field, thus effecting arbitrarily complex actuations. These actuations include the bending and even twisting of a free body when the magnetic particles form chiral domains. A theory of torsional deformations is given for both helically and circularly structured magnetic composites, and a good agreement with experiment was found.

Microstructural analysis of the field-cured gels demonstrates that particle chaining is the source of susceptibility anisotropy, with the chain direction in the axis of maximum susceptibility. The macroscopic actuation of the shape-memory gel is attributed to the tendency of these chains to align with the actuating field.

Finally, magnetic alginate that exhibits biomimetic dynamics was created in the form of a butterfly. This butterfly exhibits a hopping motion when subjected to a sequence of uniform field pulses. Because a uniform field cannot exert a body force, this hopping is surprising, but the friction of the butterfly with the surface beneath it seems to be the symmetry-breaking factor responsible for the motion.

Future directions include the possibility of more complex shape-memory geometries in both the off-field and on-field states, perhaps to explore the direction of biomimetic dynamics more fully or to pursue applications.

## Experimental Section

### Materials

Sodium alginate (empirical formula, (C_6_H_7_O_6_Na)_*n*_) of molecular weight 10,000–600,000 g/mol (PanReac AppliChem, USA) was used as the polymer material for the preparation of the hydrogels. Calcium carbonate (CaCO_3_), d-glucono-δ-lactone (GDL), and calcium chloride (CaCl_2_) were purchased from Sigma-Aldrich (Burlington, MA, USA). As the magnetic phase, we used silica-coated iron powder (Fe-CC) purchased from BASF (Germany). This powder consisted of spherical microparticles. For the preparation of the molds in which the hydrogels were cross-linked, we used polylactic acid (PLA) supplied by Smart Materials 3D (Spain) or a silicone elastomer (liquid silicone and catalyst) supplied by Gran Velada (Spain).

### Preparation of the Molds Used as Containers during Hydrogel Cross-Linking

PLA molds were prepared by 3D printing (Creality Ender-3 and Creality Ender-3 Pro) using PLA as the printing material. In this way, we prepared different molds, as shown in Figure 3, for the curing of the hydrogels. Additionally, we 3D-printed another PLA mold (not shown here) for curing of the silicone mold. For the silicone mold, we mixed liquid silicone with the catalyst at the weight proportion of 95:5 and placed the mixture in the PLA mold to allow curing. To avoid pinching of the silicone mold when twisting and thus obtaining a homogeneous torsion, we incorporated in the parallelepiped walls of the silicone mold, at the beginning of the curing step, a coiled wire that gives support to the final mold (Figure 3).

### Preparation of Actuators

The magnetic actuators consisted of magnetic hydrogels based on an alginate polymer and iron particles, which in some cases were also combined with nonmagnetic alginate hydrogels. For the preparation of magnetic alginate hydrogels, we prepared water solutions of sodium alginate at a concentration of 1% w/w, to which we added proper amounts of iron powder to obtain the desired concentration of magnetic particles in the final hydrogels (Table 1). We induced the ionic cross-linking of the alginate polymer by adding calcium ions by using CaCO_3_ as a source of these ions. To this aim, for each 5 mL of alginate solution, we added 7.5 mg of CaCO_3_ and 26.7 mg of GDL, and the resulting mixture was vortexed until homogeneity was reached. In water, GDL hydrolyzed to gluconic acid, which caused a slow dissolution of CaCO_3_, and thus a slow liberation of the calcium ions. Immediately after homogenizing the mixture, we poured it in appropriate molds, and the curing step was initiated. This was common for all magnetic hydrogels, but then two separate protocols were used depending on the type of actuators, as described in what follows.

#### Samples Prepared in PLA Molds

In this case, when 2 h of the curing step elapsed, we added to the hydrogel sample the same volume of a 45 mM solution of CaCl_2_ (as the sample that was initially poured to the mold) to strengthen the hydrogel cross-linking, and the sample was left overnight for complete curing. For preparing actuators under a magnetic field, the magnetic field was generated with the help of permanent magnets (Figure 3) and maintained from the beginning to the end of the curing step. The worm-like actuator (Figure 3) also contained parts of nonmagnetic hydrogel, which were prepared similarly but without the addition of magnetic particles.

#### Samples Prepared in Silicone Molds

For torsional actuators, we used flexible silicone molds instead of rigid PLA molds. For these samples, immediately after pouring the mixtures in the mold, it was twisted 90° in the clockwise direction (top wall with respect to the bottom wall) (Figure 3h), and a homogeneous magnetic field of 13.15 kA/m was applied, either parallel or perpendicular to the main axis of the parallelepiped sample, using a pair of Helmholtz coils. In this case, no solution of CaCl_2_ was added. When the samples approximately achieved the gel point (20 min after the onset of the curing step), the applied magnetic field was switched off, and the silicone mold was twisted counterclockwise θ_*m*_ = 90° or θ_*m*_ = 180°, and the sample was left overnight for complete curing in this configuration, thus obtaining four different distributions of particle aggregates in the final hydrogels—see Supporting Information Videos S2, S3, S4, and S5. Therefore, in this case, two separate steps of curing took place. The first one was with the sample under a homogeneous magnetic field and twisted 90° (clockwise) and the second one without a magnetic field with the sample twisted θ_*m*_ = 90° or θ_*m*_ = 180° (counterclockwise) with respect to the first step of curing (Figure 3h).

### Mechanical Characterization of Hydrogels

For mechanical characterization of the hydrogels, we used a Discovery HR-1 rheometer (TA Instruments, USA) equipped with a parallel plate geometry of 40 mm of diameter for oscillatory shear tests and a two-clamp geometry for static tensile tests. All measurements were carried out at room temperature (25 °C). For oscillatory shear tests, the samples had a cylindrical shape with a diameter of 40 mm and a height of 1 mm, and we measured the viscoelastic moduli as a function of the shear strain amplitude at a fixed frequency of 1 Hz, as well as a function of frequency for a fixed shear strain amplitude of 0.3% within the linear viscoelastic region. For each set of experimental conditions, three different samples were measured, and the corresponding mean values and standard errors are provided in this work. Results are shown in the Supporting Information Figure S7 and as observed are typical gel-like samples, with the storage modulus (*G*′) considerably larger than the loss modulus (*G*″) within the linear viscoelastic region, and values of both *G*′ and *G*″ are weakly dependent on the frequency of oscillation within this region.

For static tensile tests, we prepared bone-shaped samples and measured the length of the sample as a function of the applied force with a constant rate of 100 μ/s of increasing sample length. From these measurements, we obtained the Young’s modulus, *E*, by the following equation
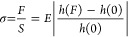
9where σ is the normal stress, *F* is the normal force applied by the upper clamp, *S* is the initial cross-sectional surface of the sample, and *h*(0) and *h*(*F*) are, respectively, its initial length and its length for a given applied force *F*. For each set of experimental conditions, three different samples were measured, and the corresponding mean values and standard errors are provided in Supporting Information Table S1.

For torsion tests, circularly structured samples 90° were prepared, with a height of 35 mm and a square section 10 mm wide. The sample was cured directly in a structure that allowed direct mounting to the two-clamp geometry of the Discovery HR-1 rheometer, so that the reliability of the measurements was ensured without any slippage. This structure consisted of two caps and an intermediate piece that fit into the caps, closing the interior space for curing the sample (Supporting Information Figure S8). Once the sample was cured, the intermediate part was removed, and the two caps were fixed to the two-clamp geometry of the rheometer for torsional experiments (Supporting Information Figure S8a,b). Subsequently, the sample was subjected, at room temperature, to an increasing ramp of torsion angle (steps of 10°) starting in the nontwisted position up to 40° of torsion. Each torsional angle was maintained for 30 s, and the residual angle was recorded for 30 s after the torsional stress was removed. The experiment was repeated for three different samples, and the corresponding mean values and standard errors are provided in this work.

### Analysis of the Behavior of the Actuators at the Microscale and Magnetic Properties

The behavior of the magnetic actuators at the microscale was assessed using a TomoTu laboratory X-ray microtomography (μCT) setup developed by the Chair of Magnetofluiddynamics, Measuring and Automation Technology, TU Dresden, Germany. The setup was based on commercially available components such as a nanofocus X-ray tube XS160NFOF (GE Sensing & Inspection Technologies, Wünsdorf, Germany), a flat plane detector Shad-o-Box 4K EV (Rad-Icon Imaging Corp., Waterloo, Canada), and a set of manipulators for aligning and rotating the sample or a specific cell with the sample. The system has already successfully proven itself for several years for the microstructure analysis of various magnetic composites, see e.g., refs (56) and (57). Within the current work, this system allowed scanning of the hydrogels to obtain information about the disposition of the magnetic particles dispersed within them. The 1440 radiographs of each sample in consideration were taken for every tomography data set with an angular increment of 0.25°, an accelerating voltage of 90 kV, an electron current of *I* = 170 μA, and an exposure time of 2 s. For the application of magnetic fields during the X-ray microCT measurements, a cell based on two moving magnets was used, so that the sample was placed at a middle distance between them. The magnetic field strength was changed by controlling the distance between the magnets. The reconstruction of the 3D images from the obtained radiographs was developed at TU Dresden and based on the FDK algorithm software package. Graphical visualization was carried out with VGStudio Max 2.1 (Volume Graphics GmbH).

Magnetization curves of the samples studied in the microCT experiments were obtained using a Lake Shore 7407 vibrating sample magnetometer (Lake Shore Cryotronics, Inc. USA) calibrated with a standard nickel sphere. All magnetization curves were measured at room temperature *T* = 23 °C, and the sample averaging period used for obtaining the magnetic moment was set to 1 s pt^–1^.

### Analysis of the Actuation Behavior

For characterization of the actuation behavior of the samples, we used an experimental setup consisting of two equal coils placed in an approximate Helmholtz configuration, with their symmetry axis vertically oriented; see Supporting Information Figure S9a,b for a photo of the whole magnetic control equipment. Each coil had an internal diameter of 24 cm and consisted of 2000 turns of copper wire of a diameter of 1.8 mm. The internal surfaces of the coils were separated by 55 mm in all experiments, except for twisting actuators, in which case the separation was 85 mm (Supporting Information Figure S10). We simulated the magnetic field generated by the coils using COMSOL Multiphysics (Supporting Information Figure S10). As observed, the field was quite homogeneous in the working region placed around the midpoint between the two coils. For actuation experiments, the samples were placed at a midpoint between the two coils, submerged in water, for which we used a transparent plastic box or small Petri dish as the container. By being submerged in water, the ambient temperature was better regulated than in air, dehydration of the actuator was prevented, and adhesion of actuators with surfaces was hindered, which allowed focusing on the pure response of the actuator to the applied magnetic field. The actuators were subjected to stepwise increasing and decreasing ramps of applied magnetic field. In some specific experiments, alternating cycles of application and removal of the magnetic field were used. In all cases, each value of the field was maintained for a time long enough to ensure the stationary response of the actuator. The behavior of each actuator was monitored by photograph and video recording. Each experimental condition was reproduced for at least three different samples to ensure significance of the results presented in this work. Representative pictures and videos, as well as mean values and standard deviations of the observed behaviors, are provided in this work.

## Data Availability

The data underlying this study are openly available in Figshare at https://doi.org/10.6084/m9.figshare.24466021.

## Supporting Information Available

Butterfly locomotion using pulses of *H* = 0/25 kA/m (MP4)
Simulation of the curing process in circularly structured magnetic composites 90° (MP4)
Simulation of the curing process in circularly structured magnetic composites 180° (MP4)
Simulation of the curing process in helically structured magnetic composites 90° (MP4)
Simulation of the curing process in helically structured magnetic composites 180° (MP4)
Circularly structured and helically structured samples responding to magnetic fields (MP4)
Change of flow direction using a circularly structured 180° actuator (MP4)
Torsion and recovery (MP4)
120 pulses of *H* on/off (MP4)
Actuation behavior of planar actuators cured under a vertical magnetic field, displacement along the *x* axis of the planar actuator imitating a butterfly, rigid plastic channel used in the fluidic application, residual deformation vs torsional deformation in torsional experiments, stability study of the actuators, measured susceptibilities and effective susceptibilities of composites of carbonyl iron particles structured by a uniaxial field, rheological characterization of different types of gels used to make the actuators, scheme of the structure used in the torsional experiments with the rheometer, experimental setup of actuation experiments, COMSOL simulation of the magnetic field generated by the coils used in actuation experiments, Young’s Modulus of the magnetic hydrogels prepared in this work, and development of the theoretical models presented in this work (PDF)
