# Supplementary material for: Fabrication and Actuation of Magnetic Shape-Memory Materials
Source: ACS Appl Mater Interfaces. 2023 Nov 4;15(45):53017–30. doi: 10.1021/acsami.3c14091 (PMC10658454; doi:10.1021/acsami.3c14091)
Supplement: Supplementary file 10 — am3c14091_si_010.pdf [file am3c14091_si_010.pdf]

Supporting Information

Fabrication and actuation of magnetic shape-memory materials

Francisco J. Vazquez-Perez[1,2], Cristina Gila-Vilchez[1,2], Alberto Leon-Cecilla[1,2], Luis Álvarez de Cienfuegos[2,3], Dmitry Borin[4], Stefan Odenbach[4], James E. Martin[5], Modesto T. Lopez-Lopez*[,1,2]

[1]Universidad de Granada, Departamento de Física Aplicada, C.U. Fuentenueva, Granada, E-18071, Spain
[2]Instituto de Investigación Biosanitaria ibs.GRANADA, Avda. de Madrid 15, Granada, E-18012, Spain
[3]Universidad de Granada, Departamento de Química Orgánica, Unidad de Excelencia Química Aplicada a Biomedicina y Medioambiente, C. U. Fuentenueva, Granada, E-18071, Spain
[4]Chair of Magnetofluiddynamics, Measuring and Automation Technology, Technische Universität Dresden, George-Bähr-Strasse 3, Dresden, 01069, Germany
[5]Sandia National Laboratories, Albuquerque, New Mexico 87059, USA

*E-mail: modesto@ugr.es

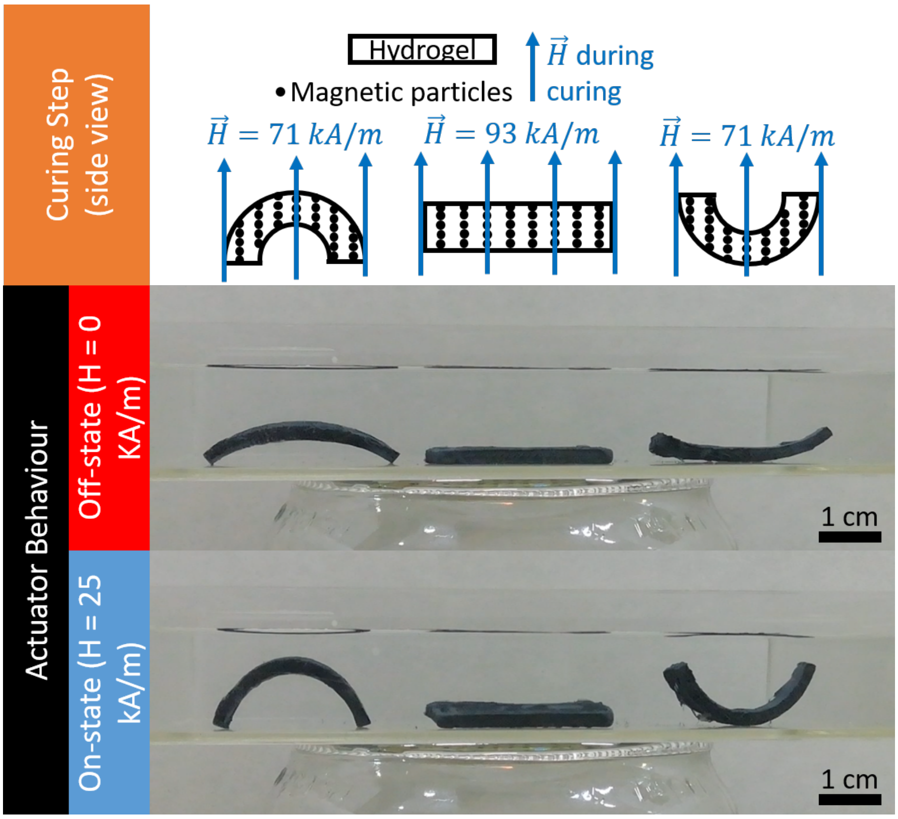

Figure S1: Actuation behavior of planar actuators cured under a vertical magnetic field. The top row presents sketches of the curing step, with indication of the shape kept during curing, the applied vertical magnetic field, and sketches of the expected distributions of magnetic particles (Fe-CC, represented by black dots). The middle row and the bottom row respectively show photographs of the magnetic hydrogels in the absence of applied magnetic field (off-state) and presence of a vertically applied magnetic field (on-state). Each column corresponds to a different hydrogel, cured under different conditions, as indicated in the top row.

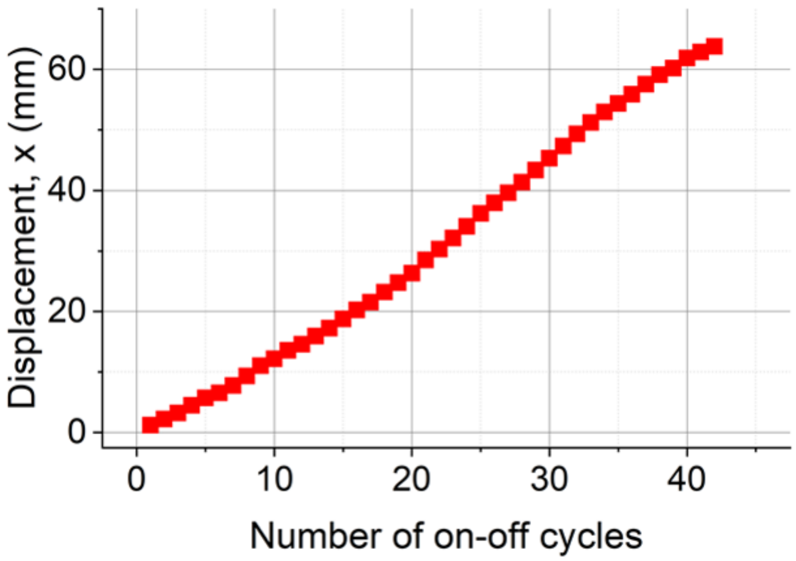

Figure S2: Displacement along the x axis of the planar actuator imitating a butterfly as a function of number of on-off cycles

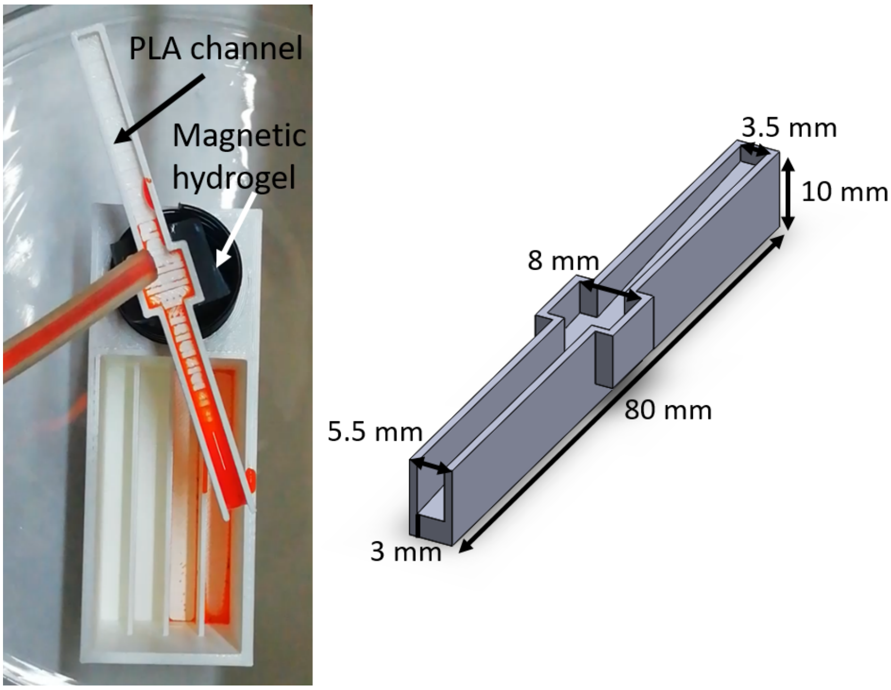

Figure S3: Rigid plastic (PLA) channel fixed to the upper surface of the hydrogel by gluing.

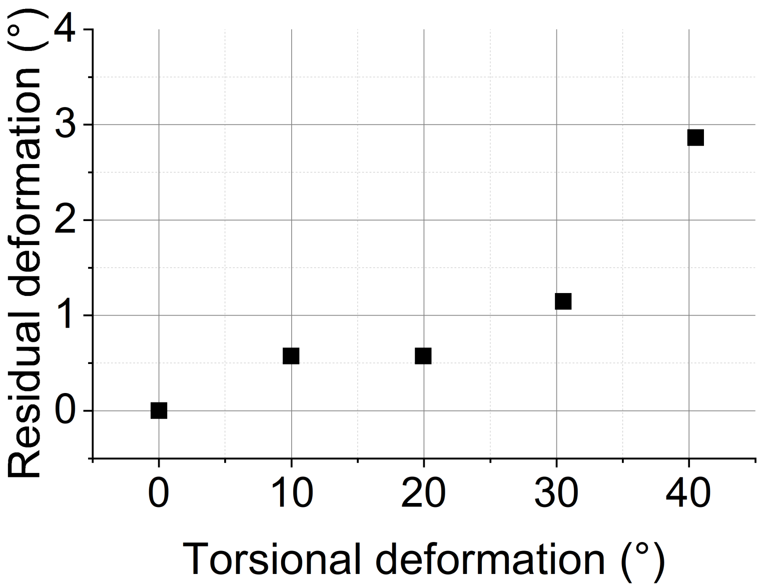

Figure S4: Residual deformation resulting from applying a torsional deformation to the hydrogel.

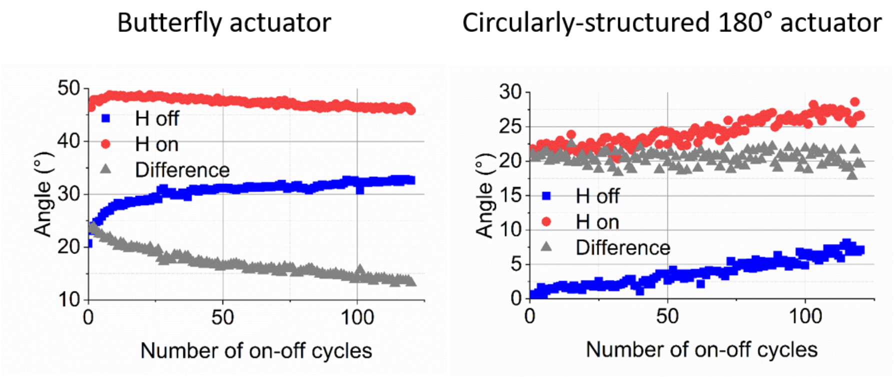

Figure S5: Deformation and recovery for the butterfly actuator and circularly-structured 180° actuator during 120 cycles of magnetic field on/off. The magnetic field was applied for 5 seconds and the actuator rested for 5 seconds in each cycle.

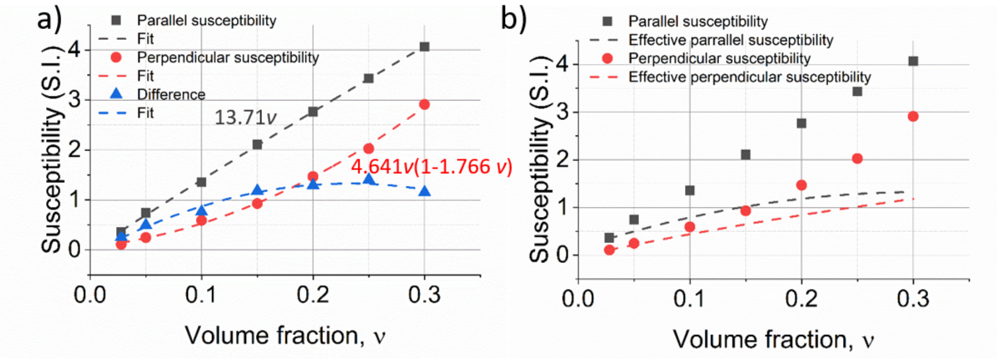

Figure S6: (a) The measured susceptibilities of composites of carbonyl iron particles structured by a uniaxial field. (b) The effective susceptibilities of cylindrical composites of carbonyl iron particles structured by a uniaxial field are considerably lower than the susceptibilities, a fact that reduces the actuation of the sample by about a factor of three. The difference in the effective susceptibilities maximizes at about 15 vol.% particles. Data of susceptibilities reprinted with permission from [James E. Marin, Eugene Venturini, Judy Odinek, and Robert A. Anderson, APS Phys. Rev. E 61, 2818-2830, 2000]. Copyright (2000) by the American Physical Society.

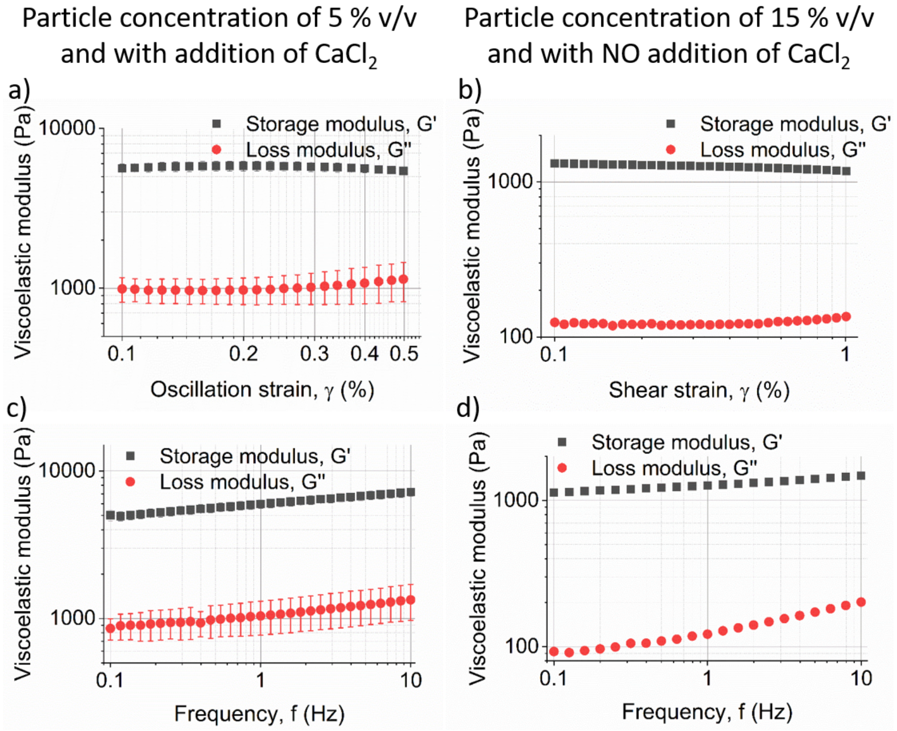

Figure S7: Rheological characterization of the different types of gels used to make the actuators. Graphs a) and b) correspond to shear strain amplitude sweeps for a constant frequency of 1 Hz. Graphs c) and d) correspond to frequency sweeps for a constant deformation amplitude of 0.3 %.

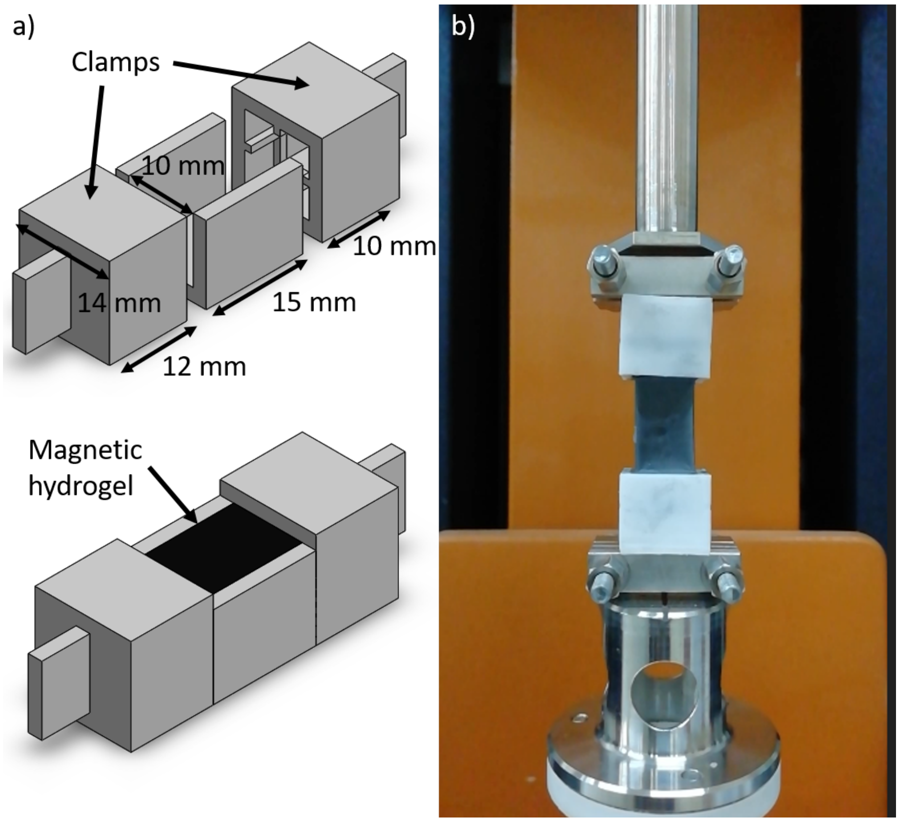

Figure S8: a) Scheme of the different parts of the mold used for the torsional experiment in the Discovery HR-1 rheometer, and scheme of the mold with the magnetic hydrogel inside. Image b) corresponds to the sample fixed in the rheometer by the two clamps in the extremes.

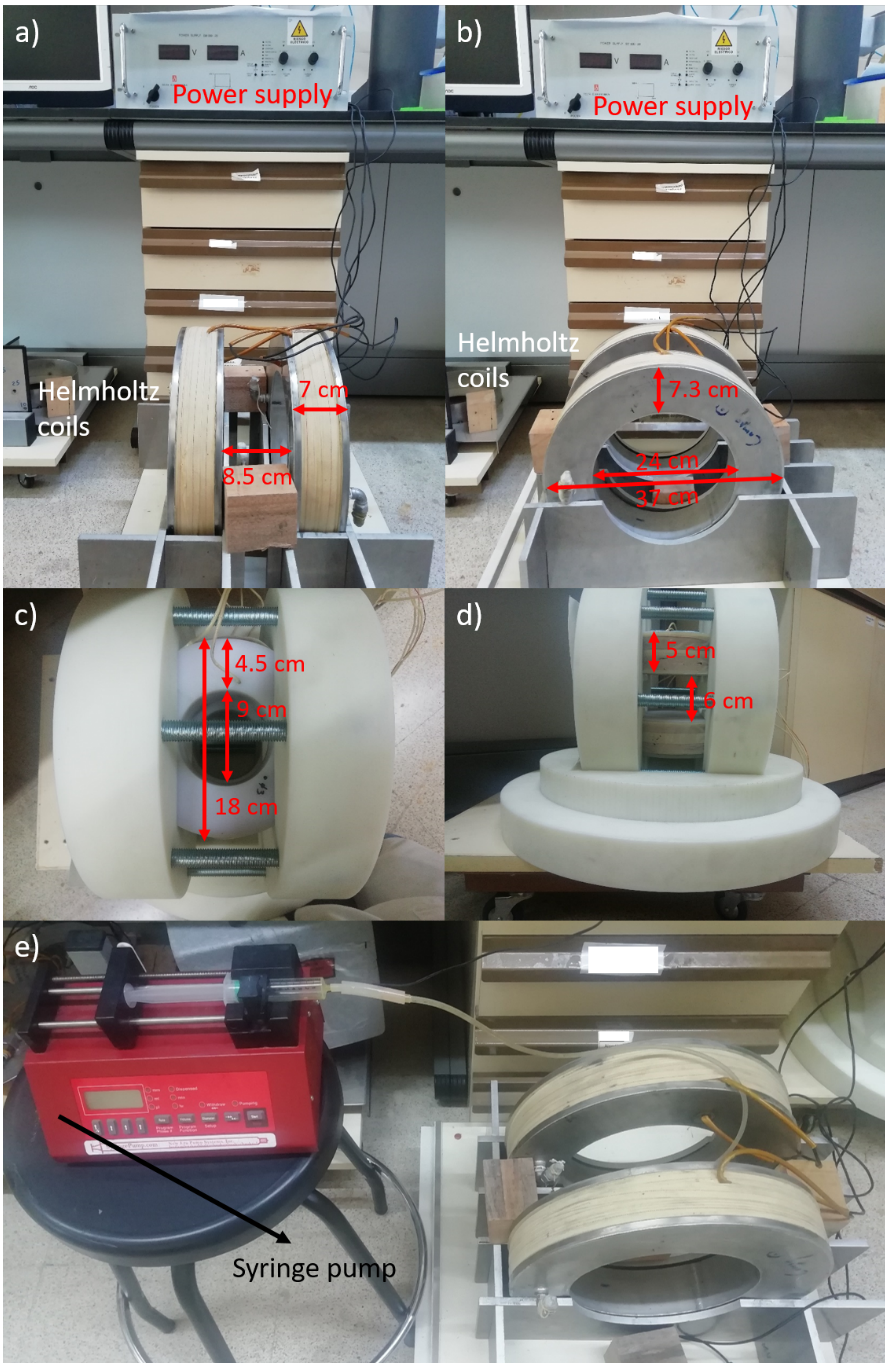

Figure S9: Pictures a) and b) correspond to the experimental set-up used in most of the actuation experiments with different perspectives of the electromagnetic coils. Pictures c) and d) correspond to different perspectives of the electromagnetic coils used in the butterfly stability experiments. Picture e) corresponds to the experimental set-up used for the fluidic application.

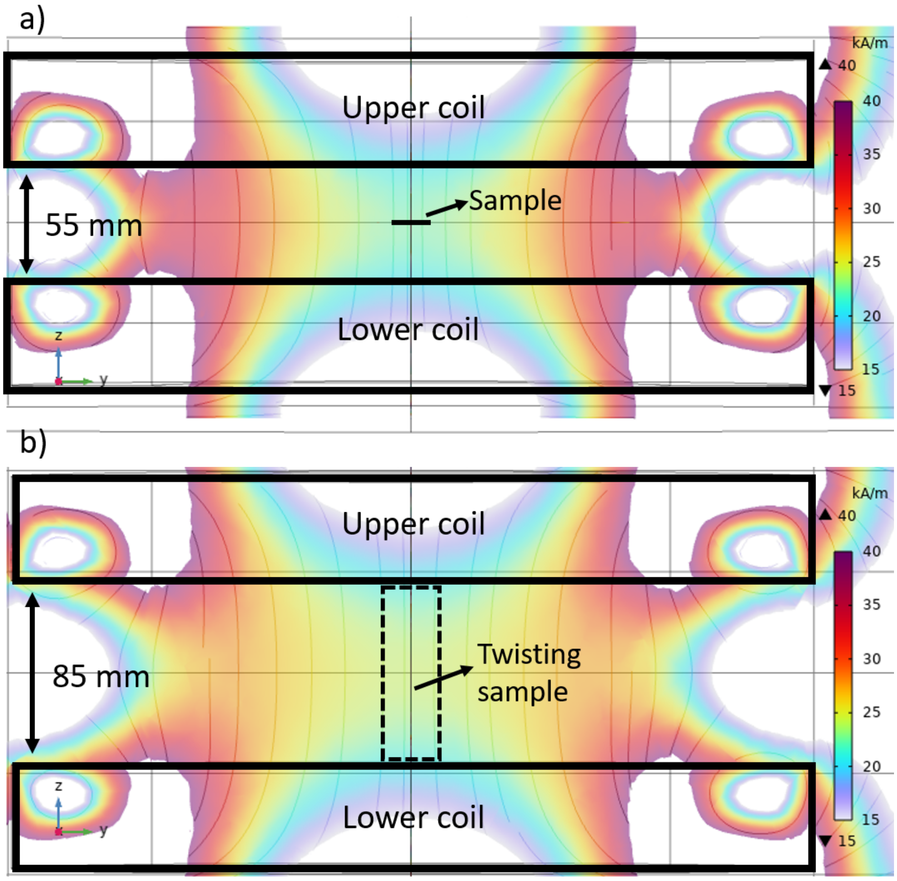

Figure S10: Comsol simulation of the magnetic field generated by the coils used in actuation experiments. Each coil consisted of 2000 turns of copper wire of 1.8 mm of diameter. Internal surfaces of the coils were separated by 55 mm (a) and by 85 mm (b).

Table S1: Young's Modulus of the magnetic hydrogels prepared in this work.

Sample	Particle concentration (% v/v)	$CaCl_2$ addition	Young's Modulus, E (kPa)
Disc, cross, worm, rectangular, semi-circular, S-shape, Butterfly	5	Yes	104 ± 4
Circularly-structured and helically-structured samples	15	No	95 ± 6

1 Magnetic energy of circularly-structured magnetic composites

Let $\chi_{\|}$ and $\chi_{\perp}$ be the composite susceptibilities parallel and perpendicular to the field-structuring direction. The composite polarizes due to its internal macroscopic field, which is given by $H_{int} = H_0 - nM$, where n is the demagnetization factor for a field applied normal to a cylinder, H_0 is the applied field, and M is the composite magnetization. The product nM accounts for the field produced by the composite magnetization, which opposes the applied field. The demagnetization factor is $n = 0.5$ for a field applied normal to a high aspect ratio cylinder but is generally somewhat less than this for typical laboratory samples. The composite magnetization parallel to the chains is given by $M_{\|} = \chi_{\|} H_{int}$ in the S.I. system, and the same is true for the perpendicular component, so

$$\begin{aligned} M_{\|} &= \frac{\chi_{\|}}{1 + n\chi_{\|}} H_0 \equiv A_{\|} H_0 \\ M_{\perp} &= \frac{\chi_{\perp}}{1 + n\chi_{\perp}} H_0 \equiv A_{\perp} H_0 \end{aligned} \tag{1}$$

For a field applied along the x axis (Figure 9).

$$\begin{aligned} \overrightarrow{M}_{\|} &= A_{\|} H_0 \cos(\theta)[\cos(\theta)\widehat{x} + \sin(\theta)\widehat{y}] \\ \overrightarrow{M}_{\perp} &= A_{\perp} H_0 \sin(\theta)[\sin(\theta)\widehat{x} + \cos(\theta)\widehat{y}] \end{aligned} \tag{2}$$

The magnetostatic energy of the composite is $U = -\frac{1}{2}V\mu_0\overrightarrow{M} \cdot \overrightarrow{H_0}$ where μ_0 the permeability of vacuum, $V = \pi R^2$ L is the volume of the composite in terms of its radius R and length L. This gives

$$U = -\frac{1}{2}V\mu_0 H_0^2[A_{\|}\cos^2(\theta) + A_{\perp}\sin^2(\theta)] \tag{3}$$

This expression shows that the energy is minimized at zero chain angle as long as $A_{\|} > A_{\perp}$.

In the composites of interest here, the orientation of the particle chains changes by an angle θ_m from one end of the composite to the other. The chain angle relative to the applied field can thus be written as $\theta = \theta_0 + \theta_m \times (z/L)$, where the cylindrical axis is along the z axis. The issue we address here is what is the orientation of this cylinder when a field is applied along the x axis. The preferred orientation will be that which minimizes the magnetostatic energy.

Integrating the magnetic energy of the cylinder along its length gives

$$U = -\frac{V\mu_0 H_0^2}{2\theta_m}\int_{\theta_0}^{\theta_0+\theta_m}[A_{\|}\cos^2(\theta) + A_{\perp}\sin^2(\theta)]d\theta \tag{4}$$

where we have used $dz = Ld\theta/\theta_m$. Completing the integrals gives

$$U = -\frac{V\mu_0 H_0^2}{2}\left\{\frac{1}{2}(A_{\|} + A_{\perp}) + \frac{1}{4\theta_m}(A_{\|} - A_{\perp})[\sin(2\theta_0 + 2\theta_m) - \sin(2\theta_0)]\right\} \tag{5}$$

Because $A_{\|} > A_{\perp}$ this energy is minimized when $\theta_0 = -\theta_m/2$ or $180 - \theta_m/2$, a result that is intuitive. The two solutions are simply because the magnetic susceptibility of a chain is symmetric under inversion. This minimum energy is just

$$U = -\frac{V\mu_0 H_0^2}{4}\left\{(A_{\|} + A_{\perp}) + (A_{\|} - A_{\perp})\frac{\sin(\theta_m)}{\theta_m}\right\} \tag{6}$$

2 Magnetic energy of helically-structured magnetic composites

The volume distribution of the cylinder is $V(r)dr = 2\pi rLdr$ and the integral of this over the interval $[0, R]$ is the cylinder volume $\pi R^2 L$. This distribution can be written in terms of the chain angles as $V(\phi)d\phi = \frac{1}{2\pi}l^2 L \times \tan(\phi)\sec^2(\phi)d\phi$. The composites we fabricate have fairly small chain angles, so to a good approximation we can write this distribution as

$$V(\phi)d\phi = 2\phi_m^{-2}V\phi d\phi \tag{7}$$

where the normalization is such that the integral over the interval $[0, \phi_m]$ is the composite volume V. This approximation greatly simplifies the final expressions, but is

not necessary, as the required integrals would still be analytic. In all that follows, this small angle approximation is carried through.

The energy in a magnetic field of a magnetic material is $U = -\frac{1}{2}\mu_0 \overrightarrow{M} \cdot \overrightarrow{H}_0$, where M is the magnetization. As a function of the chain angle, this expression is

$$U = -\frac{1}{2}\mu_0 H_0^2 [A_\parallel \cos^2\phi + A_\perp \sin^2\phi] \tag{8}$$

where $A_\parallel = \chi_\parallel/(1 + n\chi_\parallel)$ and $A_\perp = \chi_\perp/(1 + n\chi_\perp)$. Here n is the demagnetization factor for the cylinder when the field is applied parallel to its cylindrical axis, $\chi_\parallel$ is the susceptibility of the composite in S.I. units parallel to the chains and $\chi_\perp$ is the perpendicular susceptibility.

The demagnetization factor accounts for the fact that the composite polarizes due to its macroscopic internal field, not the applied field. This macroscopic field can be considerably smaller than the applied field, since the applied field is opposed by surface polarization (surface "magnetic monopoles"). The magnitude of this demagnetizing factor depends greatly on the sample geometry and the composite susceptibility, but it is in the range from 0 to 1, the latter for a field applied normal to a sheet, the former for a field applied parallel to the long axis of a fine needle. The 1947 Osborne paper [1] gives a closed-form formula for the demagnetization factor of the general ellipsoid, which is independent of the susceptibility of the material of which it was composed. This case is analytic because the internal field is uniform. The field inside a cylinder is not uniform and so numerical methods are required and the demagnetization factor depends on the material susceptibility.

The susceptibilities of uniaxially structured composites made of carbonyl iron particles are presented as a function of particle volume fraction in [2]. These susceptibilities were measured on square rectangular composites and accurate calculations of the demagnetizing fields were made to extract the intrinsic composite susceptibilities from the measured susceptibilities, both parallel and perpendicular to the direction of particle chaining. For particle volume fractions ν up to 0.30 the susceptibilities are accurately given by

$$\begin{aligned} \chi_\parallel &= 13.7\nu \\ \chi_\perp &= 4.674\nu/(1 - 1.766\nu) \end{aligned} \tag{9}$$

For a high aspect ratio cylinder n will be small and the product of this times the susceptibilities should be small compared to unity if the particle loading is not too large.

The total energy of the composite in the field is $U = -\mu_0 V H_0^2 g(\phi_m)$, where

$$g(\phi) = \phi^{-2} \int_0^\phi [A_\parallel \cos^2\phi' + A_\perp \sin^2\phi']\phi' d\phi' \tag{10}$$

Completing the integrals gives

$$g(\phi) = \frac{1}{4}(A_\parallel + A_\perp) + h(\phi)(A_\parallel + A_\perp) \tag{11}$$

where

$$h(\phi) = \phi^{-2}\left[\frac{1}{4}\phi\sin(2\phi) + \frac{1}{8}\cos(2\phi) - \frac{1}{8}\right] \tag{12}$$

Expanding the trigonometric terms shows this function is not singular at zero.

The change in the magnetic energy of the composite upon torsional deformation is given by $\Delta U(\phi) = -\mu_0 H_0^2 V \Delta g(\phi)$ where

$$\Delta g(\phi) \equiv g(\phi) - g(\phi_m) = (A_\parallel - A_\perp)[h(\phi) - h(\phi_m)] \cong (A_\parallel - A_\perp)h'(\phi_m)(\phi - \phi_m) \tag{13}$$

The derivative is

$$h'(\phi) = \left(\frac{1}{2}\phi^{-1} - \frac{1}{4}\phi^{-3}\right)\cos(2\phi) - \frac{1}{2}\phi^{-2}\sin(2\phi) + \frac{1}{4}\phi^{-3} = -\frac{1}{2}\phi + O(\phi^3) \tag{14}$$

which again is not singular at $\phi = 0$, though it appears to be.

References

[1] Osborn, J. A. Demagnetizing Factors of the General Ellipsoid. *Phys. Rev.* **1945**, *67*, 351–357.

[2] Martin, J.; Venturini, E.; Odinek, J.; Anderson, R. Anisotropic Magnetism in Field-Structured Composites. *Phys. Rev. E* **2000**, *61*, 2818–2830.
